# The gut microbiome in early life predicts malaria susceptibility

**DOI:** 10.3389/fcimb.2026.1769376

**Published:** 2026-06-23

**Authors:** Christopher L. Dutton, Madison Follis, Jenny Munaweera, Felicien Masanga Maisha, Connie J. Mulligan, Julie M. Moore

**Affiliations:** 1 Department of Biology, University of Florida, Gainesville, FL, United States; 2 Department of Anthropology, University of Florida, Gainesville, FL, United States; 3 Genetics Institute, University of Florida, Gainesville, FL, United States; 4 Genetics and Genomics PhD Program, University of Florida, Gainesville, FL, United States; 5 HEAL Africa Hospital, Goma, Democratic Republic of Congo; 6 Department of Infectious Diseases & Immunology, College of Veterinary Medicine, University of Florida, Gainesville, FL, United States

**Keywords:** Democratic Republic of Congo, early life, gut microbiome, infant, malaria, susceptibility

## Abstract

**Background:**

Despite intensive international efforts and broad implementation of control and prevention efforts, malaria continues to take a devastating toll on the most vulnerable populations, especially infants and young children. Emerging data support an important role for gut microbiome disruption in exacerbating, and potentially contributing to, adverse outcomes in malaria in young children. Less well understood are the role of the gut microbiome in early infancy in determining malaria susceptibility and how malaria exposure may impact gut microbial communities during this highly dynamic and sensitive period of microbiome development.

**Methods:**

To address these gaps, we recruited mother-infant dyads at birth in malaria-endemic eastern Democratic Republic of Congo. Infant fecal samples collected at six weeks, and at three, six and 12 months of age, as well as at passive malaria sick and post-treatment visits, were subjected to full length 16S rRNA sequencing.

**Results:**

Significant differences in relative abundance of a number of bacterial species distinguished those infants who never had a malaria visit from those who did, and those malaria episodes resulted in gut dysbiosis. Classifier analysis with Boruta selection revealed preliminary predictive capacity of the six-week fecal microbiome for malaria susceptibility through the first year of life, with a modest signal partially intertwined with bednet use. Healthy gut-associated *Bifidobacterium breve* and its metabolic partner *Cutibacterium avidum*, along with *Megasphaera micronuciformis* were associated with malaria resistance, whereas bacteria previously associated with pathogenic processes, including *Streptococcus salivarius, Klebsiella pneumoniae*, and *Rothia mucilaginosa*, associated with malaria susceptibility.

**Conclusions:**

These results provide the first evidence that gut microbial composition in early infancy is associated with subsequent malaria susceptibility. These associations, if confirmed in larger cohorts, may inform future investigation of microbiome-targeted strategies to support resistance to malaria in early life.

## 1 Introduction

Malaria is a devastating disease that impacts the lives of millions of people every year. According to the World Health Organization (WHO), the global malaria risk is increasing, with more than 280 million cases and an estimated 610,000 deaths being reported in 2024 (World Health Organization, 2025). Sub-Saharan Africa bears the most significant burden, with the Democratic Republic of Congo (DRC) having the second highest burden (12.5%) of disease and accounting for 11.1% of all malaria deaths (World Health Organization, 2025). Pregnant women, their infants and young children are at the highest risk for malaria morbidity and mortality. It is estimated that more than one third of all pregnancies in the WHO Africa Region were affected by malaria in 2024, resulting, through maternal anemia and placental damage and dysfunction (Desai et al., 2007; Hartman et al., 2010; Chua et al., 2021), in 411,000 neonates with low birth weight (World Health Organization, 2025). Children under the age of five years account for 75% of all malaria deaths in this region (World Health Organization, 2025).

A number of strategies have been implemented to provide protection against the devastating effects of malaria. These include intermittent preventive drug treatment of malaria in pregnant women, seasonal malaria chemoprevention, mass distribution of insecticide-treated bednets, indoor insecticide spraying, dissemination of malaria vaccines, and application of rapid diagnostic tests and combination anti-malarial drug therapies at the community level. Such interventions have collectively shown significant results, including an overall reduction in malaria cases by 2.3 billion and prevention of 14 million deaths since 2000 (World Health Organization, 2025). Substantial gains have been made in preventing deaths among children younger than five years; the proportion of all deaths attributable to this age group dropped from 86.7% in 2000 to 73.7% in 2023 (World Health Organization, 2024). However, the burden of disease remains significant, and, in light of recent increases in cases, continued work to identify strategies that can further mitigate the impact of malaria on vulnerable populations is warranted.

A potential new strategy is manipulation of the gut microbiome. There are numerous examples of “healthy” gut microbiota being linked to positive health outcomes, including for infectious diseases (Denny et al., 2016; Waldman and Balskus, 2018). For example, studies in endemic populations and in controlled human trials recently identified links between specific bacteria, as well as short-chain fatty acid metabolism in gut microbiota, and protection against typhoid fever (Ashton et al., 2025). Evidence is accumulating that modulation of the gut microbiota, and naturally occurring gut microbial community members, significantly influence vaccine-induced immune responses to respiratory pathogens (Xue et al., 2025). In malaria, associations between gut microbial composition and prospective risk for symptomatic and asymptomatic malaria infection have been identified in Malian children (Yooseph et al., 2015; Van Den Ham et al., 2024). In addition, significant differences in gut microbial composition have also been proposed to influence malaria outcome (mild asymptomatic disease versus severe malarial anemia) among African children (Mandal et al., 2021; Mandal et al., 2023; Bednarski et al., 2025). Direct demonstrations of the impact of the gut microbiota on severity of malaria infection in inbred (Villarino et al., 2016; Mandal et al., 2021; Mandal et al., 2023) and outbred (Morffy Smith et al., 2019) mice have lent credence to the possibility of manipulating the gut microbiota toward a protective phenotype (Yilmaz et al., 2014; Villarino et al., 2016; Moore and Morales Aparicio, 2022; Mandal and Schmidt, 2023), though to date the argument for a pathogenic and disease-driving role for specific gut taxa is more definitive (Mandal et al., 2021; Mandal et al., 2023; Bednarski et al., 2025). Nonetheless, studies are few that examine gut microbiota in the context of prospective risk for malaria, and how early development of the infant gut microbiome influences the risk for malaria is understudied (Mandal et al., 2019). Finally, severe malaria has been found to induce gut dysbiosis in mouse models (Mooney et al., 2015; Taniguchi et al., 2015; Denny et al., 2019) and in children older than six months of age (Bednarski et al., 2025), but evidence for this in early infancy is lacking (Mandal et al., 2019).

To fill these gaps in knowledge, a prospective cohort of infants recruited at birth in malaria-endemic eastern DRC was followed for the first year of life. Fecal samples collected at four follow-up visits from six weeks to one year as well as at unscheduled “sick” and post-treatment visits with a malaria diagnosis were subjected to full length 16S rRNA sequencing. To our knowledge, this is the first longitudinal study of infant gut microbiota over the first year of life in relation to malaria susceptibility. The results support for a role for malaria infection in inducing gut dysbiosis and confirm associations between components of the gut microbiota and prospective risk for symptomatic malaria, with gut microbial composition at six weeks of age showing preliminary predictive capacity for malaria infection during the first year of life.

## 2 Materials and methods

### 2.1 Ethics statement

This study was performed in accordance with human subjects research guidelines and regulations for the United States and the Democratic Republic of the Congo (DRC) and was governed by protocols approved by the University of Florida (Project #IRB202001503) and ethics committees in the DRC (Université Libre des Grands Lacs (ULPGL/Goma) and HEAL Africa Hospital, where recruitment was performed). Written, informed consent was obtained from all participants.

### 2.2 Participant recruitment and consenting

Participant recruitment and consenting for the mother-infant dyads recruited for this study have been described in detail elsewhere (Dutton et al., 2023). In brief, parturient women were recruited at HEAL Africa hospital, Goma, DRC from March to October 2020. Inclusion criteria were singleton, uncomplicated, and vaginal delivery; no apparent infection or underlying medical condition present as determined by medical history and physical examination; and willingness to bring the infant to the hospital for regularly scheduled follow-up visits and for symptomatic illness in the infant. Fifty-two women were enrolled. Five mother/infant dyads were lost to follow up, resulting in a final study sample of 47 mothers and infants.

Following their provision of written, informed consent, medical histories, demographic and behavioral data, semi-structured interviews, and surveys were collected from mothers within one day of delivery as described (Dutton et al., 2023). Participants were invited to return to HEAL Africa Hospital at regular intervals (six weeks, three months, six months and one year) for infant follow-up, as well as to return to the hospital for symptomatic illness in the infant (“sick visits”). For such visits resulting in the need for treatment of a malarial infection, follow-up post-treatment visits were scheduled for the infant to be brought back to the hospital one week later to confirm resolution of the infection.

Demographic and behavioral information were utilized to generate a malaria risk score, based on self-reported use of bednets (recorded separately for mother and infant), maternal antimalarial drug use (0=yes, 1=no) and malarial infection (0=no, 1=yes) during pregnancy, current (0=no, 1=yes) or past (0=no, 0.5=yes) malaria case in the household, and house window covering. Mothers’ bednet use was scored as 0=always/just at night or 1=sometimes/never (as at enrollment) and infant bednet use was recorded at each visit, scored as 0-1 (0=always/just at night +1=sometimes/never; averaged for three-month, six-month and one-year follow-up responses). Window covering was scored as 0=glass/plastic/glass and curtain and 1=open or curtains only.

### 2.3 Sample collection and processing

Fecal samples were collected from infants at regularly scheduled follow-up visits (six weeks, three months, six months and one year) as well as at “sick visits” in which the baby tested positive for malaria and one-week post-treatment visits. At sick visits, infants were evaluated by HEAL Africa staff, and diagnoses and medications prescribed (including antibiotics and antimalarial treatments) were recorded. Malaria was diagnosed by light microscopy on Giemsa-stained thick blood smears of heel stick or fingerprick blood. Infants were treated with artemether alone or in combination with lumefantrine, and, in one case, with quinine. Fecal samples were collected using the OMNIgene Gut OMR-200 collection kit (DNAGenotek, Kanata, ON, Canada) and processed for full length bacterial 16S rRNA gene sequencing as described (Dutton et al., 2023). In brief, DNA was extracted from fecal samples with the QIAamp PowerFecal Pro DNA extraction kit (Qiagen, Germantown, MD), quantified and quality-checked, and then aliquoted into randomized 96-well plates. Samples were sent to the University of Illinois Roy J. Carver Biotechnology Center for 16S rRNA gene library preparation and PacBio sequencing. Full-length 16S rRNA amplicons were generated, converted to libraries, and sequenced twice on a PacBio Sequel IIe in circular consensus mode.

### 2.4 Digital polymerase chain reaction

Digital PCR (dPCR) was performed on the Applied Biosystems QuantStudio Absolute Q Digital PCR System (ThermoFischer Scientific). Genomic DNA samples collected at 6 weeks (6W) were diluted 1:10 by combining 1 µL of DNA with 9 µL of nuclease free water. Each dPCR reaction was prepared in a final volume of 10 µL consisting of 2 µL of 1X Absolute Q DNA Digital PCR Master Mix, 0.5 µL of 1X custom TaqMan dPCR assay, 6.4 µL of nuclease free water, and 1.1 µL of diluted genomic DNA. Primers were used for amplification of PanBacteria (Ba04930791_s1), K. pneumoniae (Ba04932083_s1), and Bifidobacterium (Ba07922592_s1). Reactions were dispensed by loading 9 µL of the dPCR mixture onto MAP16 digital PCR plates, followed by the addition of 15 µL of Absolute Q Isolation Buffer to each well. Thermal cycling was carried out using the manufacturer’s recommended conditions: an initial denaturation at 96°C for 10 minutes, followed by 40 cycles of denaturation at 96°C for 5 seconds, and annealing/extension at 60°C for 15 seconds.

### 2.5 Statistical analysis

The ability of the malaria risk score to predict infant parasite density was analyzed by multiple linear regression and plotted in R, considering infant sex, birthweight and age at the time of infection as covariates. Categorical data/proportions were compared by Fisher’s exact or C^2^ test. Continuous variables were compared by student’s t test for normally distributed data and by Mann Whitney test for nonparametric data. Malaria and stress score data were plotted as a function of dichotomized infant malaria outcome and analyzed with two-tailed unpaired t test in GraphPad Prism software (V10.4.0).

R programming language was used for all microbiome analyses as described (Dutton et al., 2023). Briefly, phyloseq (McMurdie and Holmes, 2013) and microeco (Liu et al., 2025) packages supported analyses of alpha and beta diversity, and differential abundance, controlling for infant sex and antibiotic use, was assessed with ANCOM-BC (Lin and Peddada, 2020) version 2.10.1, with bednet use included as an additional covariate in a sensitivity analysis at six weeks. SplinectomeR (Shields-Cutler et al., 2018) version 0.1.0 was applied for longitudinal assessments of relative abundance. Additionally, pairwise comparisons between relative abundances of genera and species in malaria-resistant and malaria-susceptible infants at six weeks of age were performed using Differential Abundance Analysis by Consensus (DAR package in R) (https://bioconductor.org/packages/release/bioc/html/dar.html), which applies several statistical tests (Wilcoxon, ALDEx, DESeq2, corncob, metagenomeseq, MaAsLin, and LEFSE). Only comparisons yielding significance by at least two tests by DAR are reported as statistically significantly different.

To identify microbial features predictive of later malaria infection, we implemented a comprehensive classification pipeline using the microeco framework in R, relying initially on data from the six week time point for all infants. For each taxonomic level (Phylum through Species), six supervised learning algorithms were trained with and without Boruta-based feature selection: Random Forest, SVM (radial and linear kernels), logistic regression (GLM), k-nearest neighbors, and Naive Bayes. Data were split 75%/25% for training and testing, and models were evaluated via 5-fold cross-validation with saved class probabilities. Feature selection, where applied, used Boruta with a maximum of 200 runs and p-value threshold 0.01. Model performance metrics extracted included accuracy, sensitivity, specificity, precision, F1 score, and balanced accuracy. Classifier performance was further evaluated with repeated stratified cross-validation (10 repeats × 5-fold), leave-one-out cross-validation, and permutation testing (1,000 permutations of outcome labels). Sensitivity analyses incorporated covariates (bednet use, antibiotic exposure, infant sex, and malaria risk score) alongside microbial features. Full results for these analyses and for each model–taxonomic level combination are summarized in **Supplementary Methods File 1**.

To prioritize microbial taxa that exhibit robust, multidimensional evidence of association with malaria risk, we computed three complementary importance measures for each feature: (1) absolute Pearson correlation with the binary outcome (malaria resistance versus susceptibility; “Importance”), (2) Cohen’s d effect size quantifying standardized mean differences between groups, and (3) a Wilcoxon‐derived –log_10_(p-value) (“Wilcoxon_Importance”). Each metric captures a distinct aspect of association—linear consistency, magnitude of separation, and nonparametric significance, respectively—thereby mitigating the risk of false positives that can arise when relying on any single criterion. We standardized each measure to z-scores and summed them to generate a Composite_Score; Composite_Score = Z(Importance) + Z(Effect_Size) + Z(Wilcox_Importance). Details of model testing and selection and link to the complete analysis pipeline are provided in **Supplementary Methods File 1**.

## 3 Results

### 3.1 Infant malaria development and risk factors

Forty-seven mother-infant dyads were followed up in this study. These dyads were previously examined in a study designed to assess the impact of maternal psychosocial stressors on development of the infant gut microbiome over the first year of life (Dutton et al., 2023). For the current study, infants were assigned to one of two groups based on the occurrence of any diagnosed malaria infection during the one year follow up period, where resistant was defined as never developed symptomatic malaria, and susceptible was defined as development of at least one infection resulting in a hospital visit. Summary data for the mothers and infants, stratified by infant malaria diagnosis, are presented in **Tables 1**, **2**. Among the mothers, comparison of those whose infant went on to develop a malaria infection and those whose infant remained malaria-free revealed that the groups were similar in terms of psychosocial demographics (Dutton et al., 2023), laboratory measures at parturition, reproductive and health history, and socioeconomic factors (**Table 1**). Likewise, the newborns were similar. All were born vaginally, and birth weights, frequency of low birth weight, length at birth and percent males between the two infants groups were comparable (**Table 1**). Eighteen infants developed at least one symptomatic malaria infection that was detected through passive follow up (**Table 2**). Malaria cases were observed during the entire one year follow up period from 6 to 52 weeks, with 83% occurring in infants older than 5 months/24 weeks. Three infants had two discreet malaria episodes. **Table 2** summarizes developmental, medical and nutritional data for the infants, again stratified by malaria infection incidence, for each of the scheduled follow ups (six weeks, three months, six months and one year). Sleeping under an insecticide-treated bednet is known to significantly reduce malaria prevalence and morbidity when applied soon after birth (Muller et al., 2006). In this cohort, prevalence of consistent sleeping under a bednet was statistically significantly lower in the malaria-susceptible group (39%) relative to the malaria resistant group (78%) at the six week follow up (P = .0127). Thereafter, the groups were similar in terms of bednet use, with overall frequency declining with increasing age (**Table 2**). Antibiotics were frequently prescribed, increasing from 11% of infants at six weeks to 38% at six months. At the one year visit, significantly more malaria-susceptible infants had been treated with antibiotics since the last visit (78%) compared to the malaria-resistant infants (44%; P = .0336). Otherwise, the two infant groups were similar. Notably, most infants were exclusively breastfed during the first few months of life (98% at six weeks, 91% at three months), with rates declining at six months (33%) with the introduction of solid foods. Fever at or prior to the follow up visits (<5%) and diarrhea since the last visit (2 to 13%) were infrequent and hospitalizations were rare (<3% up to six months; 7% at one year).

**Table 1:** Descriptive characteristics of study population at enrollment stratified by infant malaria status.

Characteristic^a^	All infants (n = 47)	Malaria+ (n = 18)	Malaria- (n = 29)	*P*-value^b^
Maternal psychosocial demographics				
Age (years)	27.0 ± 7.24	27.8 ± 7.55	26.5 ± 7.13	0.56
Parity	3.02 ± 2.57	3.28 ± 2.78	2.86 ± 2.37	0.59
Married (%)	39/47 = 83.0%	15/18 = 83.3%	24/29 = 82.8%	1.0
Composite stress score	3.14 ± 1.08	3.33 ± 1.03	3.01 ± 1.11	0.33
Intent at delivery to use infant bednet	46/47 (97.9%)	18/18 (100%)	28/29 (96.6%)	1.0
Body mass index	28.0 ± 3.94	27.5 ± 4.17	28.24 ± 3.84	0.53
Maternal laboratory				
Temperature at admission	36.2 ± 0.38	36.2 ± 0.43	36.1 ± 0.35	0.47
Fever at admission^c^	0/47 (0%)	0/18 (0%)	0/29 (0%)	1.0
Antibiotics received intrapartum	45/47 (95.7%)	17/18 (94.4%)	28/29 (96.6%)	1.0
Maternal self-reported health/reproductive history				
Antenatal care visits^d^	4.29 ± 0.92 (n = 45)	4.0 ± 0.97	4.48 ± 0.85 (n = 27)	0.1
Previous miscarriage	9/47 (19.2%)	4/18 (22.2%)	5/29 (17.2%)	0.72
Previous stillbirth	3/47 (6.38%)	2/18 (11.1%)	1/29 (3.45%)	0.55
Previous post early neonatal loss	4/47 (8.51%)	1/18 (5.56%)	3/29 (10.3%)	1.0
Malaria during pregnancy	8/46 (17.4%)	2/17 (11.8%)	6/28 (21.4%)	0.46
Malaria symptoms experienced, not treated.	2/12 (16.7%)	1/4 (25%)	1/8 (12.5%)	1.0
Hospitalization for malaria during pregnancy	6/47 (12.8%)	1/18 (5.56%)	5/29 (17.2%)	0.38
Maternal malaria treatment received	10/12 (83.3%)	3/4 (75%)	7/8 (87.5%)	1.0
IPTp doses received	1.93 ± 0.50 (n = 44)	1.89 ± 0.47	1.96 ± 0.53 (n = 26)	0.64
Previous treated GI parasitism	42/47 (89.4%)	18/18 (100%)	24/29 (82.8%)	0.14
One-year antibiotic use	2/47 (4.26%)	1/18 (5.56%)	1/29 (3.45%)	1.0
Always used bednet during pregnancy	9/47 (19.2%)	4/18 (22.2%)	5/29 (17.2%)	0.72
Newborn				
Vaginal delivery	47/47 (100%)	18/18 (100%)	29/29 (100%)	1.0
Birth weight (g)	3205.8 ± 380.6 (n = 45)	3225.6 ± 342.8 (n = 16)	3184.8 ± 405.4	0.79
Low birth weight (≤2500 g)	1/45 (2.22%)	0/16 (0%)	1/29 (3.45%)	1.0
Length at birth (cm)	46.9 ± 2.05	46.3 ± 2.7	47.2 ± 1.47	0.16
Male infant (%)	25/47 (53.2%)	9/18 (50%)	16/29 (55.2%)	0.77
Household				
Own home	14/47 (29.8%)	3/18 (16.7%)	11/29 (37.9%)	0.19
House construction: concrete walls	7/46 (15.2%)	4/17 (23.5%)	3/29 (10.3%)	0.4
House construction: finished roof (tile or tin)	46/46 (100%)	17/17 (100%)	29/29 (100%)	1.0
House construction: windows (glass or plastic)	36/46 (78.3%)	11/17 (64.7%)	25/29 (86.2%)	0.14
Current household malaria case	5/47 (10.6%)	1/18 (5.56%)	4/29 (13.8%)	0.64
Previous household malaria case	12/47 (25.5%)	4/18 (22.2%)	8/29 (27.6%)	0.74
Household size	7.04 ± 3.49 (n=46)	6.88 ± 3.46 (n=17)	7.14 ± 3.56	0.81

^a^ Data are shown as number (percent) or mean ± SD unless otherwise noted (use median (interquartile range; range) where appropriate. Sample sizes are shown where missing data reduce group numbers for specific parameters, where only a subset of the group have values >0, where respondents indicated “don’t know”, or where test results were uninterpretable.

^b^ Categorical data/proportions compared by Fisher’s exact or *X*^2^ test. Continuous variables compared by student’s t test for normally distributed data and by Mann Whitney test for nonparametric data.

^c^ Fever defined as ≥38°C.

^d^ Questions answered as “more” in the questionnaire were given a value of 5.

**Table 2:** Descriptive characteristics of infants at regularly scheduled follow-up visits, stratified by infant malaria status.

Characteristics^a^	All infants (n = 47)	Malaria+ (n = 18)	Malaria- (n = 29)	*P*-value^b^
Six weeks				
Age (weeks)	5.75 ± 0.49 (n=44)	5.70 ± 0.47 (n=17)	5.78 ± 0.51 (n=27)	0.64
Height (cm)	50.9 ± 2.92 (n=45)	50.8 ± 3.86	51 ± 2.16 (n=27)	0.8
Weight (kg)	4988.89 ± 773.93 (n=45)	4928.89 ± 1046.70	5028.89 ± 541.43 (n=27)	0.71
Head circumference	38.7 ± 2.61 (n=45)	38.3 ± 2.8	39.0 ± 2.48 (n=27)	0.37
Fever at visit^c^	0/45 (0%)	0/18 (0%)	0/27 (0%)	1.0
Illness since birth	3/45 (6.67%)	1/18 (5.56%)	2/27 (7.41%)	1.0
Fever since birth	2/45 (4.44%)	2/18 (11.1%)	0/27 (0%)	0.15
Siblings with malaria since birth	0/45 (0%)	0/18 (0%)	0/27 (0%)	1.0
Infant diarrhea since birth	1/45 (2.22%)	0/18 (0%)	1/27 (3.70%)	1.0
Infant antibiotic since birth	5/45 (11.1%)	1/18 (5.56%)	4/27 (14.8%)	0.63
Infant hospitalized since birth	1/44 (2.27%)	0/17 (0%)	1/27 (3.7%)	1.0
Infant always sleeps under bednet	28/45 (62.2%)	7/18 (38.9%)	21/27 (77.8%)	0.01
Infant is breastfed exclusively	44/45 (97.8%)	17/18 (94.4%)	27/27 (100%)	0.40
Three months				
Age (months)	2.84 ± 0.42 (n=45)	2.82 ± 0.39 (n=17)	2.86 ± 0.45 (n=28)	0.80
Height (cm)	55.6 ± 4.26 (n=45)	55.5 ± 4.26 (n=17)	55.6 ± 4.33 (n=28)	0.95
Weight (kg)	6340.22 ± 728.39 (n=45)	6260.0 ± 808.64 (n=17)	6388.67 ± 686.07 (n=28)	0.57
Head circumference	41.29 ± 2.35 (n=45)	41.65 ± 2.98 (n=17)	41.07 ± 1.90 (n=28)	0.48
Fever at visit^c^	1/45 (2.22%)	0/17 (0%)	1/28 (3.57%)	1.0
Illness since last follow up	2/45 (4.44%)	0/17 (0%)	2/28 (7.14%)	0.52
Tested positive for malaria since last visit	2/47 (4.26%)	2/18 (11.1%)	0/29 (0%)	0.14
Fever since last follow up^c^	1/45 (2.22%)	0/17 (0%)	1/28 (3.57%)	1.0
Siblings with malaria since last follow up	1/45 (2.22%)	1/17 (5.88%)	0/28 (0%)	0.38
Infant diarrhea since last follow up	6/45 (13.3%)	1/17 (5.88%)	5/28 (17.9%)	0.38
Infant antibiotic since last follow up	14/45 (31.1%)	5/17 (29.4%)	9/28 (32.1%)	1.0
Infant hospitalized since last follow up	0/45 (0%)	0/17 (0%)	0/28 (0%)	1.0
Infant always sleeps under bednet	21/45 (46.7%)	10/17 (58.8%)	11/28 (39.3%)	0.23
Infant is breastfed exclusively	41/45 (91.1%)	15/17 (88.2%)	26/28 (92.9%)	0.0
Infant consumes solid foods	0/45 (0%)	0/17 (0%)	0/28 (0%)	1.0
Infant consumes water only from a tap	0/45 (0%)	0/17 (0%)	0/28 (0%)	1.9
Six months				
Age (months)	5.74 ± 0.46 (n=42)	5.6 ± 0.51 (n= 15)	5.78 ± 0.42 (n=27)	0.26
Height (cm)	62.6 ± 3.62 (n=42)	61.5 ± 3.68 (n=15)	63.2 ± 3.51 (n=27)	0.16
Weight (kg)	7881.19 ± 965.05 (n=42)	7836.0 ± 912.0 (n=15)	7906.30 ± 1009.42 (n=27)	0.82
Head circumference	43.19 ± 2.18 (n=42)	43 ± 1.81 (n=15)	43.30 ± 1.38 (n=27)	0.65
Fever at visit^c^	1/42 (2.38%)	1/15 (6.67%)	0/27 (0%)	0.36
Illness since last follow up	6/42 (14.3%)	3/15 (20.0%)	3/27 (11.1%)	0.65
Tested positive for malaria since last visit	4/47 (8.51%)	4/18 (22.2%)	0/29 (0%)	0.02
Fever since last follow up^c^	2/42 (4.76%)	1/15 (6.67%)	1/27 (3.70%)	1.0
Siblings with malaria since last follow up	1/42 (2.38%)	0/15 (0%)	1/27 (3.70%)	1.0
Infant diarrhea since last follow up	1/42 (2.38%)	0/15 (0%)	1/27 (3.70%)	1.0
Infant antibiotic since last follow up	16/42 (38.1%)	6/15 (40.0%)	10/27 (37.0%)	1.0
Infant hospitalized since last follow up	1/42 (2.38%)	0/15 (0%)	1/27 (3.70%)	1.0
Infant always sleeps under bednet	17/42 (40.5%)	7/15 (46.7%)	10/27 (37.0%)	0.74
Infant is breastfed exclusively	14/42 (33.3%)	6/15 (40.0%)	8/27 (29.6%)	0.73
Infant consumes solid foods	5/42 (11.9%)	2/15 (13.3%)	3/27 (11.1%)	1.0
Infant consumes water only from a tap	3/42 (7.14%)	1/15 (6.67%)	2/27 (7.41%)	1.0
One year				
Age (months)	11.4 ± 0.49 (n=44)	11.3 ± 0.49 (n=18)	11.4 ± 0.50 (n=26)	0.74
Height (cm)	68.1 ± 2.49 (n=44)	67.8 ± 1.66 (n=18)	68.2 ± 2.94 (n=26)	0.52
Weight (kg)	9498.96 ± 1739.14 (n=44)	9602.78 ± 1629.73 (n=18)	9426.92 ± 1839.31 (n=26)	0.75
Head circumference	46.2 ± 1.75 (n=44)	46.1 ± 1.78 (n=18)	46.3 ± 1.76 (n=26)	0.72
Fever at visit^c^	1/43 (2.33%)	1/18 (5.56%)	0/25 (0%)	0.42
Illness since last follow up	6/44 (13.6%)	4/18 (22.2%)	2/26 (7.69%)	0.21
Tested positive for malaria since last visit	15/47 (31.9%)	15/18 (83.3%)	0/29 (0%)	1.09 x 10^-9^
Fever since last follow up^c^	1/44 (2.27%)	1/18 (5.56%)	0/26 (0%)	0.41
Siblings with malaria since last follow up	2/44 (4.55%)	1/18 (5.56%)	1/26 (3.85%)	1.0
Infant diarrhea since last follow up	2/43 (4.65%)	1/17 (5.88%)	1/26 (3.85%)	1.0
Infant antibiotic since last follow up	25/43 (58.1%)	14/18 (77.8%)	11/25 (44.0%)	0.03
Infant hospitalized since last follow up	3/44 (6.83%)	2/18 (11.1%)	1/26 (3.85%)	0.56
Infant always sleeps under bednet	10/44 (22.7%)	3/18 (16.7%)	7/26 (26.9%)	0.49
Infant is breastfed exclusively	0/44 (0%)	0/18 (0%)	0/26 (0%)	1.0
Infant consumes solid foods	31/44 (70.5%)	14/18 (77.8%)	17/26 (65.4%)	0.51
Infant consumes water only from a tap	37/44 (84.1%)	14/18 (77.8%)	23/26 (88.5%)	0.42

^a^ Data are shown as number (percent) or mean ± SD unless otherwise noted (use median (interquartile range; range) where appropriate. Sample sizes are shown where missing data reduce group numbers for specific parameters, where only a subset of the group have values >0, where respondents indicated “don’t know”, or where test results were uninterpretable.

^b^ Categorical data/proportions compared by Fisher’s exact or χ^2^ test. Continuous variables compared by student’s t test for normally distributed data and by Mann Whitney test for nonparametric data.

^c^ Fever defined as ≥38°C.

**Figure 1:**
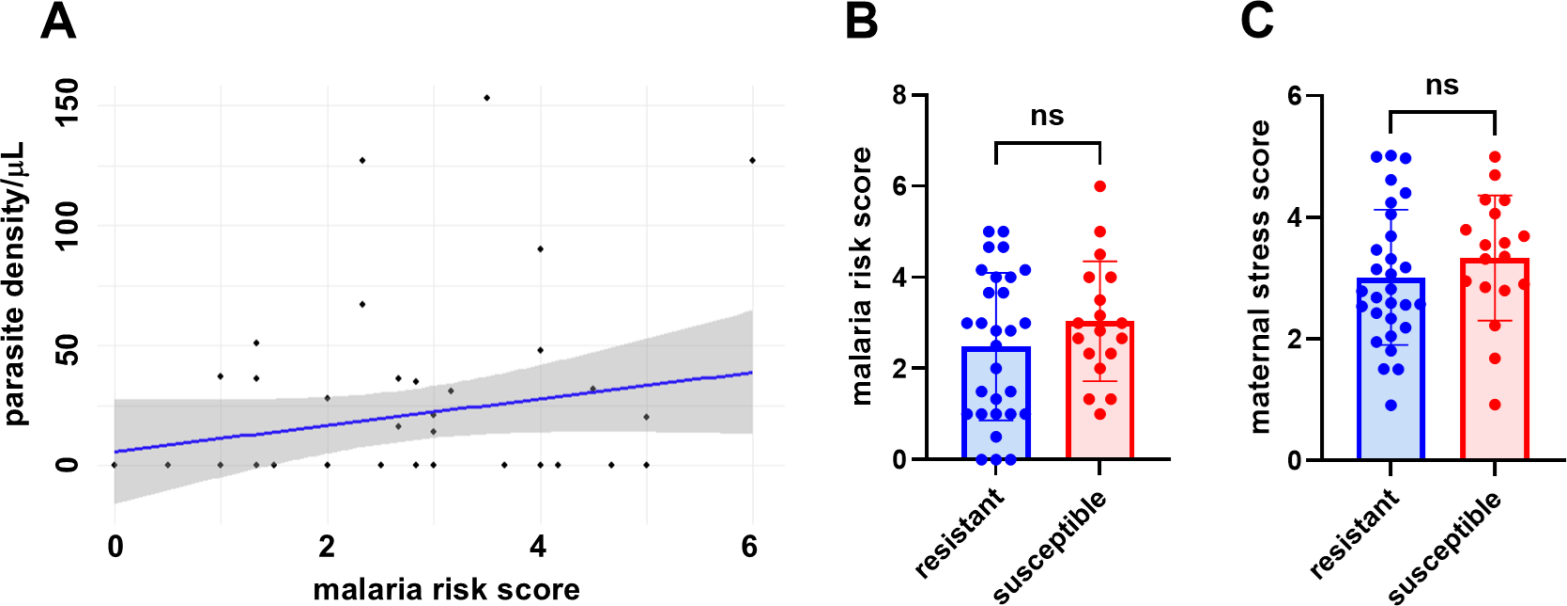
Malaria risk and maternal stress scores do not vary as a function of infant malaria susceptibility. Malaria risk score was calculated as described in the participant recruitment and consenting section. **(A)** Scatter plot of malaria risk score relative to infant parasite density with 95% confidence interval for the regression line, controlled for infant weight and sex and maternal age. **(B, C)** Infants were dichotomized based on malaria diagnosis over the one year follow up period where “resistant” identifies infants that were never diagnosed with malaria, and “susceptible” identifies infants that were diagnosed with malaria at least once during the follow up period. **(B)** Malaria risk score stratified by infant malaria status. **(C)** Maternal stress score (defined as reported [Dutton et al., 2023)] stratified by infant malaria status. ns, not significant.

Sensitivity analyses incorporating covariates into the k-NN classifier confirmed that bednet use at six weeks is an important co-predictor of malaria outcome. The microbiome-only model achieved an AUC of 0.607, while addition of bednet use increased performance to 0.787 (p < 0.0001). This is consistent with the significant difference in bednet use between groups at six weeks (39% vs. 78%, P = .0127) and indicates that the microbiome signal and bednet use are partially intertwined. Notably, the composite malaria risk score, which integrates bednet use along with other environmental exposure variables, did not differ between groups (**Figure 1B**), and its relationship with parasite density was weak and nonsignificant (adjusted R² = 0.066, P = .144). Disentangling the independent contributions of the microbiome and bednet use will require larger cohorts with sufficient statistical power to adjust for multiple covariates simultaneously.

Questionnaire information was used to assess malaria risk at the household level. Bednet use for mother and infant, self-reported anti-malarial drug use during pregnancy, maternal and household malarial cases, and environmental risk (window coverings), yielded malaria risk scores ranging from 0 to 6. In multiple linear regression modeling controlling for infant sex, birthweight and age at infection, each point increase in malaria risk score predicted an 8 parasite/μL increase (8.22 ± 3.71 (SEM), p = .0321; **Figure 1A**) and each gram increase in birthweight predicted an incremental increase in parasite density (0.30 ± 0.14 (SEM), p = .0440), though the overall model was weak and nonsignificant (adjusted r^2^ = 0.066, p = .144). Malaria risk score was not statistically significantly different between infants dichotomized to malaria-resistant and -susceptible groups (**Figure 1B**). We previously assessed multiple measures of stress in this cohort of mothers and developed a composite stress score to summarize measures of stress, anxiety, depression and trauma (Dutton et al., 2023). Like the malaria risk score, the stress score did not differ between infants based on malaria susceptibility (**Figure 1C**).

### 3.2 Development of the infant gut microbiome over the first year of life

**Figure 2:**
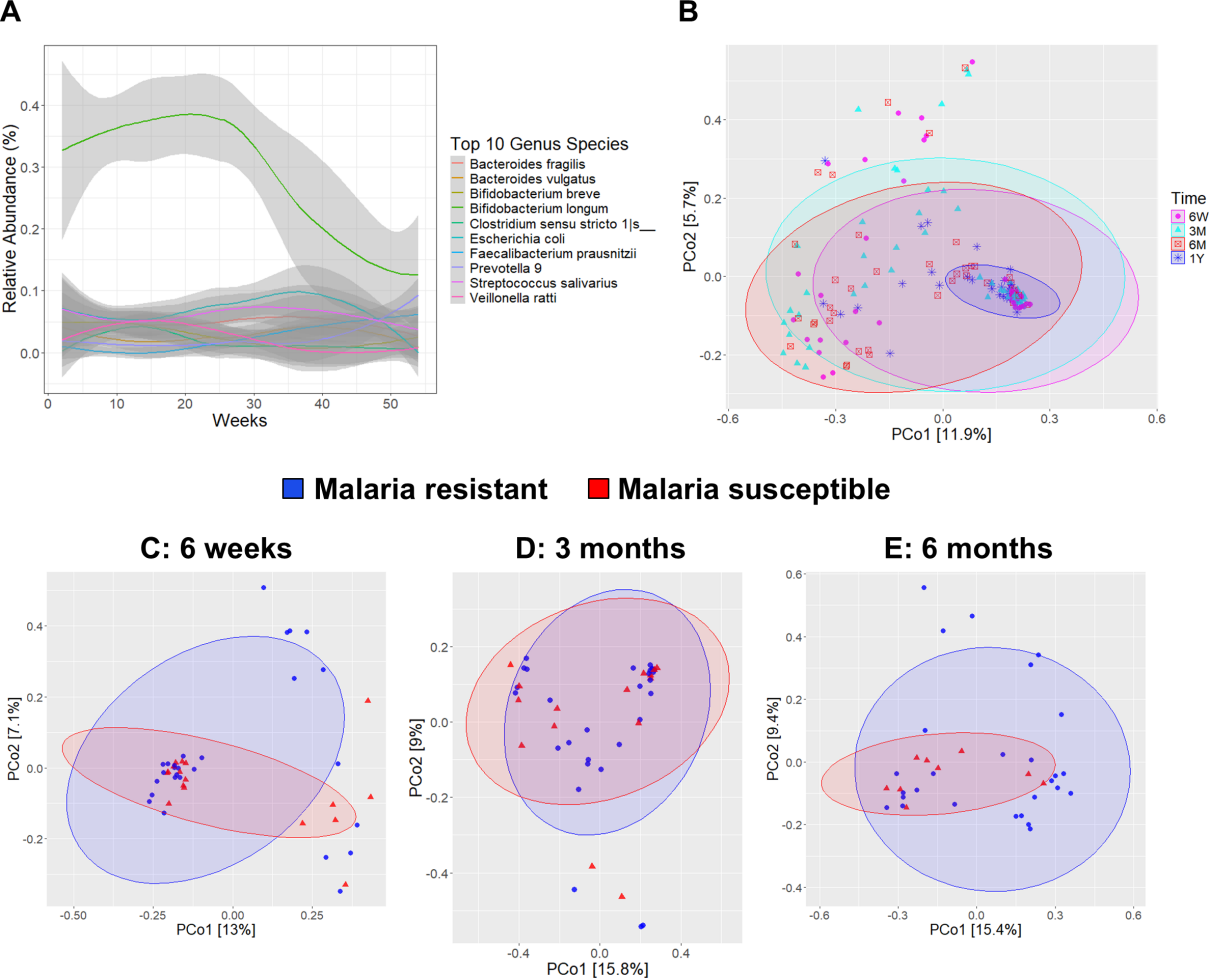
Longitudinal changes in infant fecal microbiota over the first year of life. **(A)** Longitudinal SplinectomeR analysis of gut microbiota abundance for all infants over the one year follow up period, showing the top ten most abundant species. **(B)** Principal coordinate analysis of Bray-Curtis distance for infants at the time points shown. Principal coordinate analysis of Bray-Curtis distance at **(C)** six weeks, **(D)** three months, and **(E)** six months of age reveals no differences as a function of malaria resistance or susceptibility. 6W, six weeks; 3M, three months; 6M, six months; 1Y, one year.

As previously described for the first six months of life in this cohort of infants (Dutton et al., 2023), the infant gut microbiota changed dramatically over the first year of life, with *Bifidobacterium longum* being the dominant species, representing more than 30% of all species prior to 30 weeks of age (**Figure 2A**). *Escherichia coli, Streptococcus salivarius, Bacteroides fragilis*, and *Bifidobacterium breve* were also among the most abundant genera across the first year of life, though dramatic shifts in relative abundance were evident during the follow up period. In general, a high degree of variability among infants was evident up to six months of age, with a significant contraction in within-group variance at one year (**Figure 2B**).

### 3.3 The infant gut microbiome associates with malaria susceptibility

To assess infant fecal microbial diversity as a function of malaria susceptibility with advancing age, infants were again categorized as resistant or susceptible, with data for any infant having already experienced malaria being censored from subsequent timepoints. At six weeks, three months and six months of age, microbial species richness and evenness (within group/alpha diversity) among the malaria-resistant and -susceptible infants did not differ by multiple measures (observed OTUs, Chao1, Shannon, Simpson, Inverse Simpson, and Fisher; all by Kruskal-Wallis Rank Sum Test, adjusted p > 0.05, **Supplementary Figure 1**). Likewise, between group (beta) diversity, which measures sample dissimilarity in terms of microbial taxonomic composition, was equivalent between malaria-resistant and malaria-susceptible infants at six weeks, three months and six months of age (**Figures 2C–E**).

**Figure 3:**
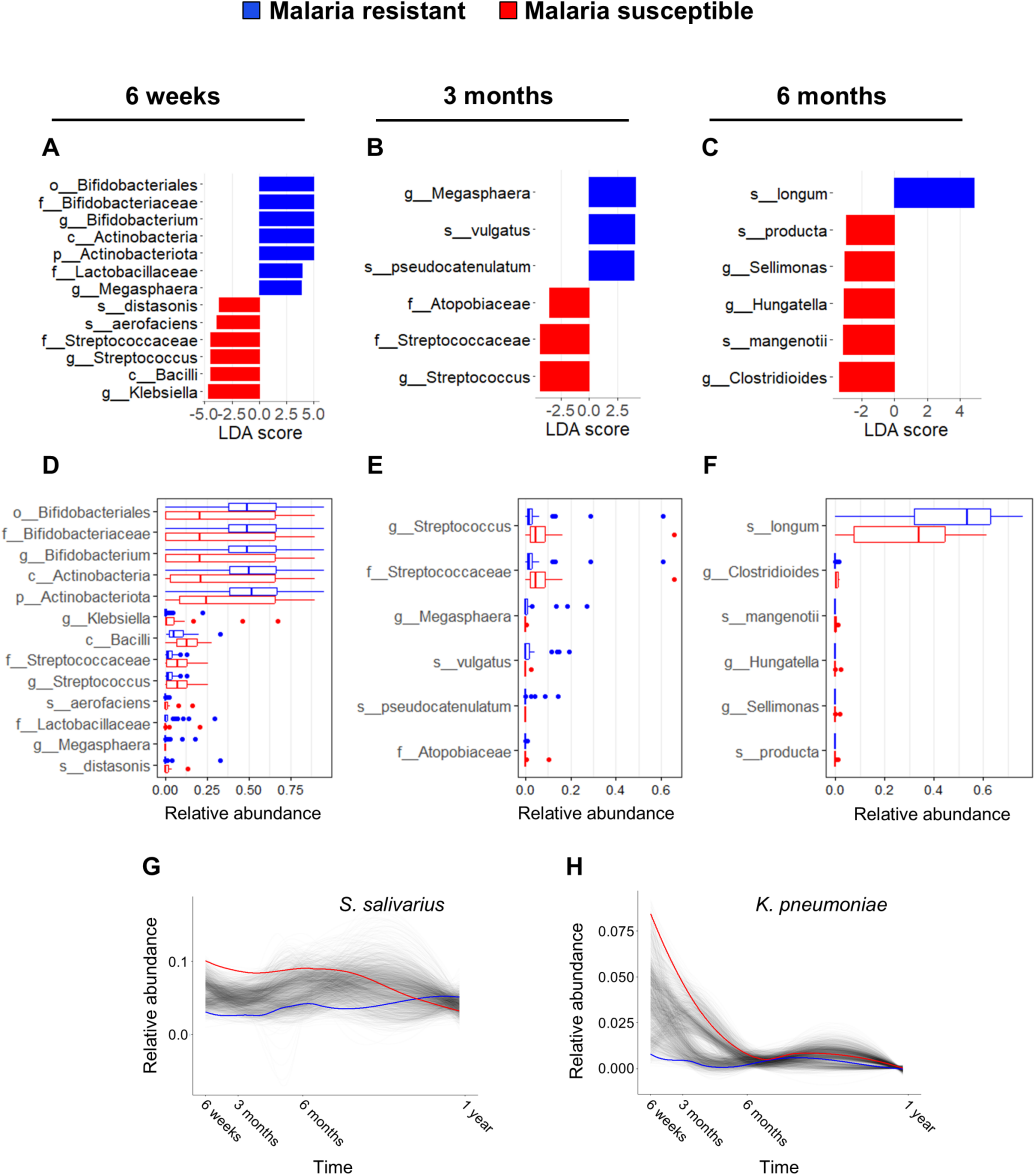
Linear discriminate analysis reveals gut microbiome features that distinguish infants on the basis of malaria susceptibility. **(A–C)** Histograms of linear discriminant analysis (LDA) reveals the most differentially abundant taxa between resistant and susceptible infants at **(A)** six weeks, **(B)** three months and **(C)** six months of age. Only taxa meeting the criteria (p < 0.05 by Kruskal-Wallis test; LDA score > 2) are shown. **(D–F)** Comparative relative abundances for the significant taxa observed in **(A)** are summarized for the same samples. In all cases, infants already diagnosed with malaria prior to the represented timepoint are censored from the analysis. **(G, H)** SplinectomeR analyses of **(G)** *S. salivarius* (p = .307) and **(H)** *K. pneumoniae* (p = .047) over the one year follow up period.

**Figure 4:**
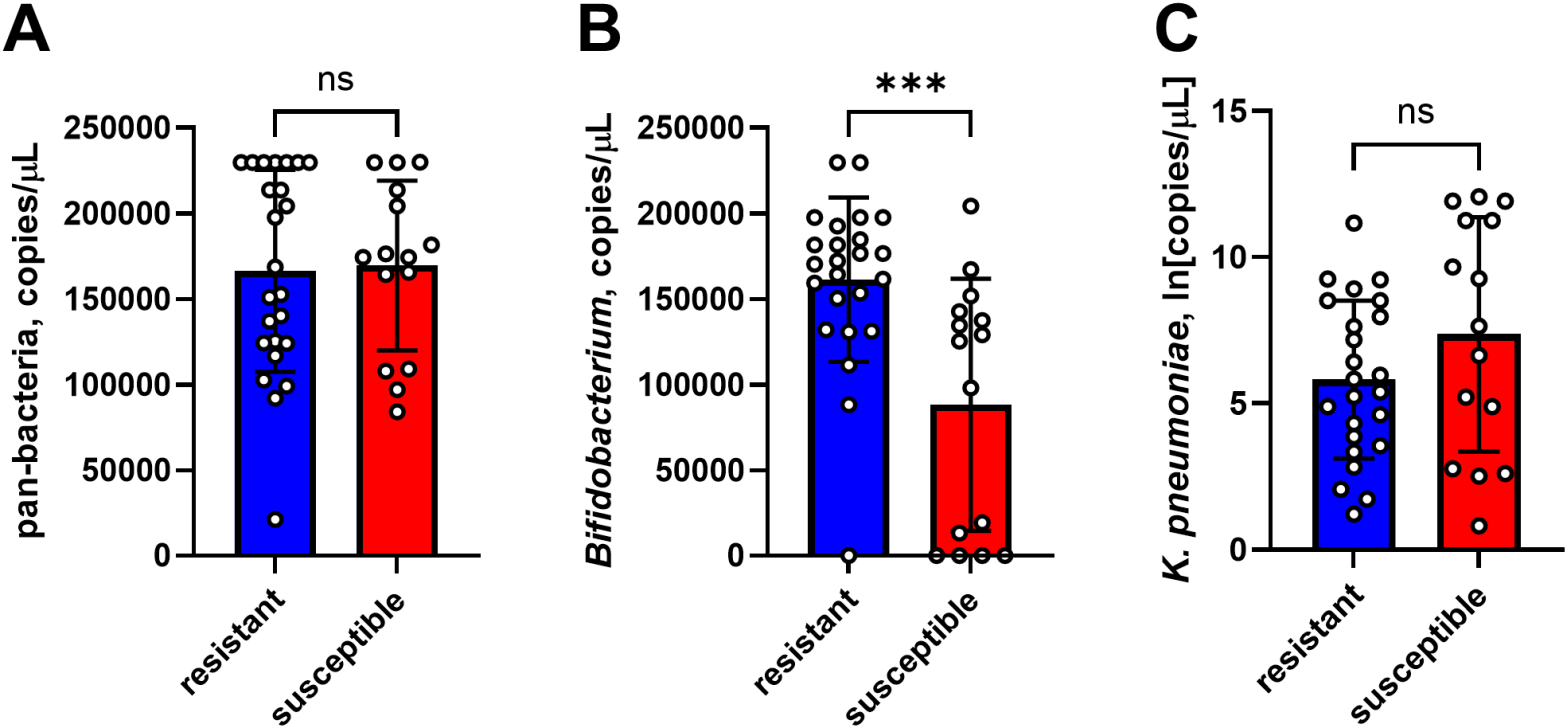
Digital PCR confirms elevated *Bifidobacterium* but unchanged *K. pneumoniae* in the gut microbial communities of malaria-resistant relative to malaria-susceptible six-week old infants. DNA samples from six-week stool samples were analyzed using **(A)** a pan-bacterial 16S rRNA primer set, as well as genus/species-specific primers for **(B)** *Bifidobacterium*, and **(C)** *Klebsiella pneumoniae* (data ln-transformed to normality). ***p = .0008. **(A, B)** Mann-Whitney U test; **(C)** two-tailed unpaired t test. Ns, not significant.

Despite the lack of observed differences in alpha and beta diversity in the infant gut microbiota dichotomized by malaria susceptibility, differences in relative abundance of discreet taxa were identified by linear discriminate analysis (LDA; **Figure 3**). Class Actinobacteria, family Lactobacillaceae and *Megasphaera* were more abundant among malaria-resistant infants at six weeks (**Figures 3A, D**) with *Megasphaera* and *Bacteroides vulgatus* being more evident in this group at three months as well (**Figures 3B, E**). Order Bifidobacteriales, family Bifidobacteriaceae and genus *Bifidobacterium* predominated in and discriminated between malaria-resistant and malaria-susceptible infants at six weeks (**Figures 3A, D**). *B. longum* was the dominant species at six months and was more highly represented in the malaria-resistant infants (**Figures 3C, F**). At three months, *B. pseudocatenulatum* was the most abundant representative of this genus among malaria-resistant infants (**Figures 3B, E**). However, Streptococcaceae and *Streptococcus* were most dominant among the differentially abundant bacteria at this time point (**Figures 3B, E**) and were more highly represented among malaria-susceptible infants (**Figure 3E**). Notably, *Streptococcus*, as well as *Klebsiella*, which are both associated with an unhealthy microbiome in infants and young children (Sarker et al., 2016; Mathew et al., 2019; Olm et al., 2019; McCartney and Hoyles, 2023), were more highly represented in six week old-infants that went on to develop malaria later in infancy (**Figures 3A, D**). To examine the extent to which these two species persist through the first year of life, SplinectomeR analysis was performed. As identified by LDA analysis, relative abundance of *S. salivarius* was relatively high early in life for malaria-susceptible infants, but this difference waned over the first year of life, yielding an overall nonsignificant difference (**Figure 3G**). *K. pneumoniae*, identified at the genus level as more abundant in malaria-susceptible children by LDA analysis at six weeks of age, showed a similar pattern of predominance in early life followed by convergence of abundance in the two groups at approximately six months of age, though, in this case, the patterns were significantly different (**Figure 3H**, p = .047). By digital PCR, abundance of *Bifidobacterium*, shown to be elevated in malaria-resistant infants at six weeks (**Figures 3A, D**) was confirmed to be higher at this time point (**Figure 4B**), against a backdrop of similar bacterial DNA concentrations in both groups of infants (**Figure 4A**). In contrast, *Klebsiella pneumoniae* rRNA copy numbers did not differ in this direct analysis (**Figure 4C**).

**Figure 5:**
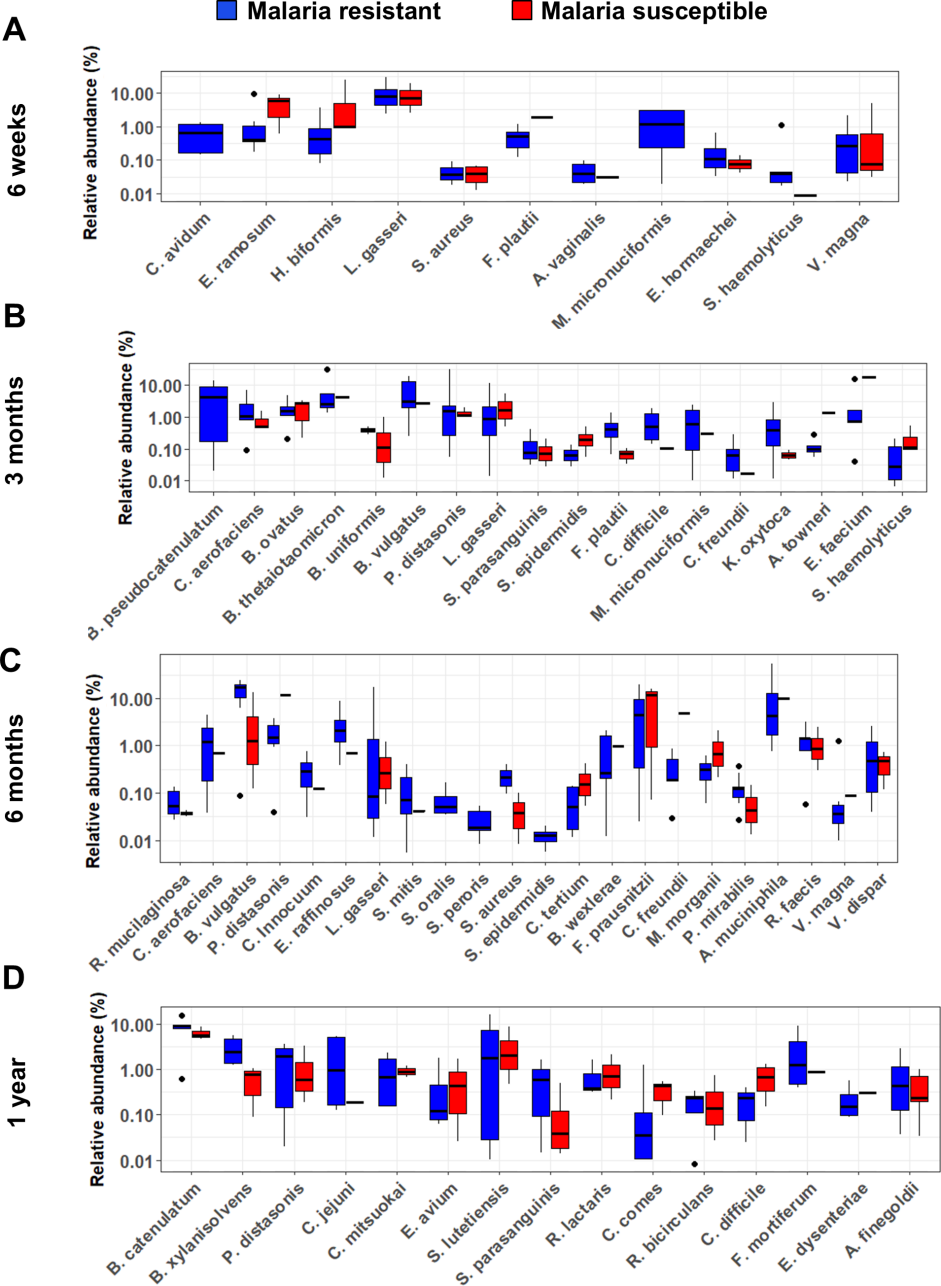
Analysis of composition of microbiomes with bias correction reveals gut microbiome features that distinguish infants on the basis of malaria susceptibility. **(A–C)** Relative abundance plots determined by ANCOM-BC, controlling for infant sex and antibiotic use, show significantly differentially abundant taxa between resistant and susceptible infants at **(A)** six weeks, **(B)** three months and **(C)** six months of age. **(D)** Data for infants at one year of age reflect groups stratified by never having had malaria (resistant) or having had malaria at any time during the follow up period (susceptible). Only taxa meeting the criteria for significance are shown.

Further characterization of differential abundance at the microbial species level was done using analysis of composition of microbiomes with bias correction (ANCOM-BC), which controlled for infant sex and antibiotic use (**Figure 5**; heat maps provided in **Supplementary Figure 2**). The one-year infant follow up sample was also assessed in this analysis, with past malaria infection being used to define the “malaria-susceptible” group (**Figure 5D**). Several of the features identified here were consistent with the LDA and digital PCR analyses, including higher relative abundance of *B. pseudocatenulatum* and *B. vulgatus* in malaria-resistant infants at three months (**Figures 3B, E**, **4B**, **5B**); the latter was also highly represented in this group at six months of age (**Figure 5C**). *Megasphaera micronuciformis* was abundant in malaria-resistant infants at six weeks and three months (**Figures 5A, B**) as observed for this genus by LDA at the same timepoints (**Figures 3A, B, D, E**). Consistent with the identification of abundant Lactobacillaceae in malaria-resistant infants at six weeks of age by LDA analysis (**Figures 3A, D**), *Lactobacillus gasseri* was similarly revealed to be abundant by ANCOM-BC (**Figure 5A**) and remained elevated in the malaria-resistant infants at three months and six months as well (**Figures 5B, C**; **Supplementary Figure 2**). Notably, the elevated abundance of *Streptococcus* observed by LDA analysis in association with malaria susceptibility at six weeks and three months (**Figures 3A, B, D, E**) was not observed by ANCOM-BC (**Figures 5A, B**).

### 3.4 Malaria in the first year of life alters relative fecal microbial diversity

**Figure 6:**
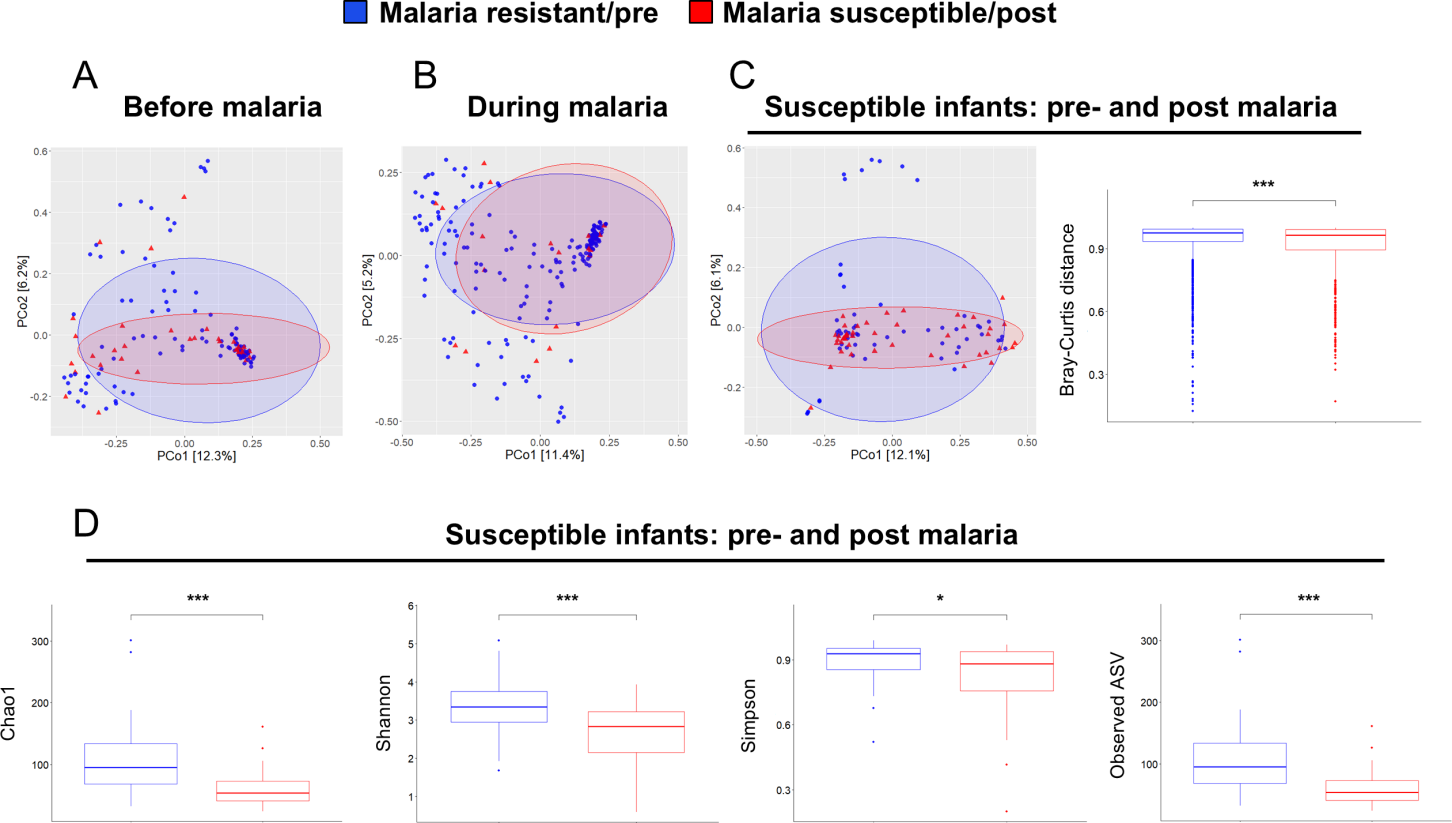
Malaria infection in infancy impacts gut microbial diversity. **(A–C)** Principle coordinate analysis of Bray-Curtis distances across the first year of life in infants resistant to malaria compared to susceptible infants at all timepoints prior to experiencing a malaria episode [**(A)**, “before malaria”], during the malaria episode [**(B)**, “during malaria”], and following the first malaria episode [**(C)**, left, “post malaria”]. [**(C)**, right] Beta diversity shown as Bray-Curtis difference and **(D)** multiple measures of alpha diversity in susceptible infants before (blue) and after (red) their first malaria infection. *p<0.05, **p<0.01, ***p<0.001.

Along with evidence of a potential protective role for the gut microbiome against malaria risk in children shown here and by others (Yilmaz et al., 2014; Yooseph et al., 2015; Van Den Ham et al., 2024), a potential dysbiotic effect on the human fecal microbiome by malaria infection has also been observed (Mandal et al., 2019; Bednarski et al., 2025). In this cohort, assessment of Bray-Curtis distance by principal coordinate analysis of all relative abundance data amongst malaria-resistant infants relative to all data from infants prior to a malaria infection revealed no differences between the groups (**Figure 6A**). Similarly, beta diversity in resistant infants relative to susceptible infant gut microbiota during their active infection was similar (**Figure 6B**). Examination of malaria-susceptible infant gut microbiota after parasitological cure relative to their pre-malaria status, however, revealed significant differences in both beta diversity (**Figure 6C**) and multiple measures of alpha diversity (**Figure 6D**).

### 3.5 The six-week infant gut microbial community is associated with and shows preliminary predictive capacity for malaria susceptibility during the first year of life

**Figure 7:**
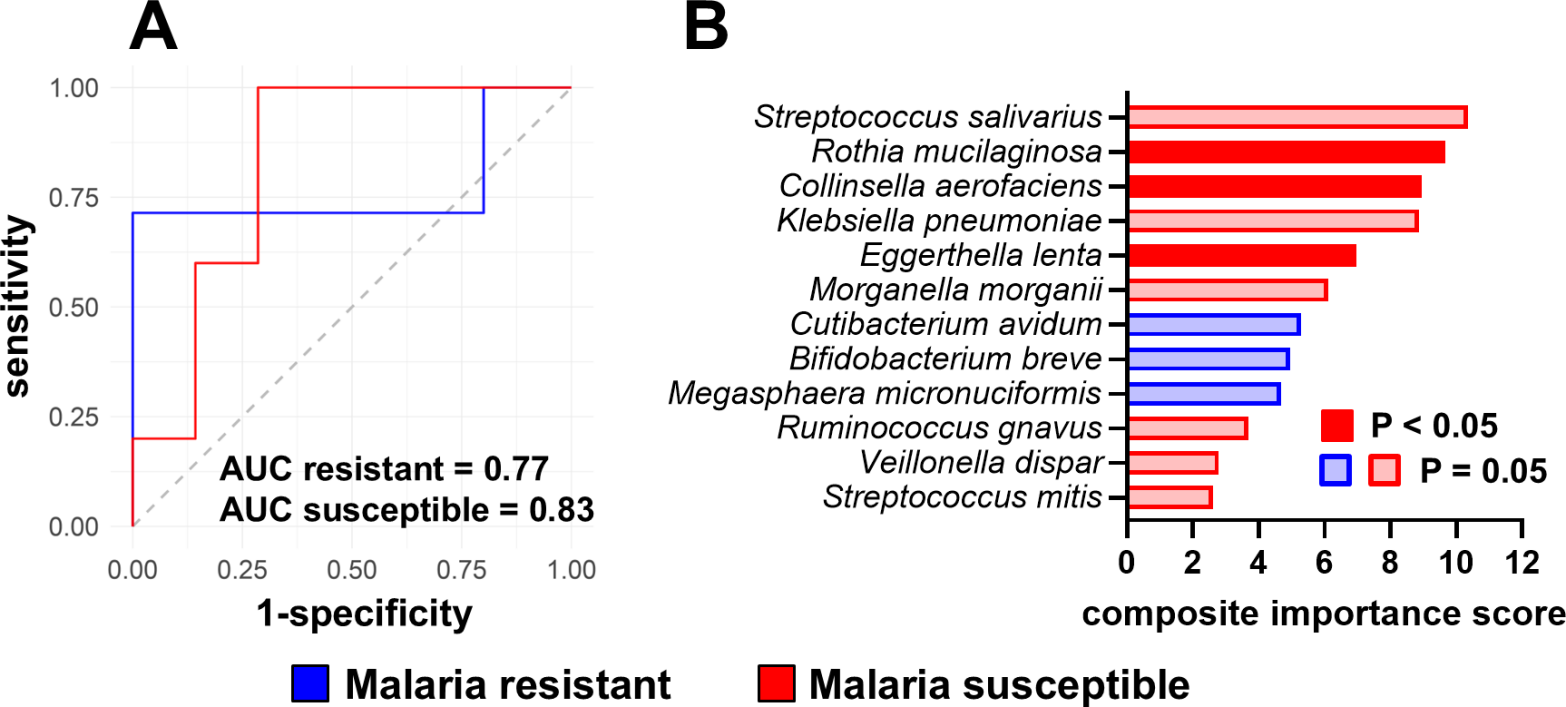
Classifier analysis shows that the six week infant fecal microbiome predicts malaria susceptibility in the first year of life. Results from k-nearest neighbors classifier analysis with feature selection at the species level in which malaria-resistant infants (who never developed malaria) were defined as “positives”. Malaria resistance features are shown in blue and malaria susceptibility features are shown in red. **(A)** Areas under the curve (AUC) with sensitivity and 1-specificity are shown for malaria-resistant and malaria-susceptible infants. **(B)** The top 12 species identified in feature importance analysis as predictive of malaria resistance and susceptibility are represented according to their composite importance score.

Given the differential abundance of fecal microbes as early as six weeks of age in infants destined to remain malaria-resistant or be malaria-susceptible during the first year of life, classifier analysis with Boruta selection was applied to identify key taxa that predict malaria resistance and susceptibility. Several analytical approaches were compared to initially interrogate the six week data set (**Supplementary Methods File 1**). The optimal classifier was achieved using k-nearest neighbors with feature selection at the species taxonomic level, yielding 90.0% balanced accuracy, 91.7% overall accuracy, 100% sensitivity, 80.0% specificity, 87.5% precision, and 93.3% F1 score. This model utilized 6 microbial features selected through Boruta feature selection from the original 144 species-level taxonomic data. The confusion matrix analysis (**Supplementary Figure 3**) revealed that the model correctly classified 9 out of 12 test samples, with 5 true positives, 4 true negatives, 1 false positive, and 2 false negatives. The overall accuracy was 75% with a Cohen’s kappa of 0.50, indicating moderate agreement beyond chance. The 95% confidence interval for accuracy ranged from 43% to 95%, with the accuracy null hypothesis value of 58% and p-value of 0.19. McNemar’s test yielded a p-value of 1.00, suggesting no significant difference in marginal frequencies of correct and incorrect classifications. Receiver operating characteristic curves for the two groups show that the model yielded adequate discriminatory power, with malaria susceptibility (AUC = 0.83) slightly outperforming malaria resistance (AUC = 0.77) (**Figure 7A**). A similar classifier analysis approach was used to assess prediction of malaria risk based on cumulative fecal microbiota data from the infants at all follow ups. This model failed at the test set step (**Supplementary Methods File 1**), perhaps due to the rapid evolution and restructuring of the infant gut microbiome over the first year of life (Ferretti et al., 2018; Stewart et al., 2018) which masked malaria-associated factors. The feature importance analysis of six-week fecal samples identified key microbial taxa most strongly associated with malaria risk classification and were ranked according to a composite importance score (**Figure 7B**). The top positive associations (higher in the susceptible group) included *Streptococcus salivarius* (composite score: 10.37), *Rothia mucilaginosa* (9.66), *Collinsella aerofaciens* (8.96), *Klebsiella pneumoniae* (8.87), *Eggerthella lenta* (6.97), and *Morganella morganii* (6.10). The top negative associations (higher in the resistant group) included *Cutibacterium avidum* (5.29), *Bifidobacterium breve* (4.95), and *Megasphaera micronuciformis* (4.68). Additional significant taxa with positive associations with malaria susceptibility included *Ruminococcus gnavus* (3.68), *Veillonella dispar* (2.79), *Streptococcus mitis* (2.63), *Lawsonella clevelandensis* (2.55), *Bacteroides finegoldii* (2.55), and *Prevotella copri* (2.55) (**Supplementary Figure 3**).

Repeated stratified cross-validation (10 repeats × 5-fold) yielded a mean AUC of 0.642 ± 0.204, with sensitivity of 0.744 ± 0.195 and specificity of 0.518 ± 0.282. Leave-one-out cross-validation produced a balanced accuracy of 62.0% (sensitivity 59.3%, specificity 64.7%, Cohen’s kappa = 0.227). Permutation testing (1,000 permutations) yielded a p-value of 0.312, consistent with limited statistical power at n = 44. Out-of-sample predictive capacity of the microbiome classifier is therefore modest. Incorporating bednet use at six weeks as a covariate improved model performance (AUC 0.607 to 0.787, p < 0.0001), indicating that the microbiome signal and bednet use are partially intertwined as predictors of malaria outcome (**Supplementary Methods File 1**). Importantly, ANCOM-BC differential abundance results at six weeks were robust to additional adjustment for bednet use, with 11 taxa retaining significance (q < 0.05), indicating that the taxon-level associations are not driven by this covariate.

Assessment of differences in relative abundance of taxa at the genus level in six-week fecal samples using Differential Abundance Analysis by Consensus (DAR; **Supplementary Table 1**) confirmed that *Streptococcus* was more highly represented in malaria susceptible infants, whereas *Bifidobacterium, Lactobacillus*, and *Megasphaera* were significantly elevated in the malaria-resistant infants. *Lacticaseibacillus* was identified as more abundant in this group as well. At the species level, two taxa identified by composite importance score as significantly different between the two infant groups, *R. mucilaginosa* and *C. aerofaciens* (**Figure 7B**), were found by DAR to be differentially abundant and significantly higher in the malaria-susceptible infants (**Supplementary Figure 4**; **Supplementary Table 2**). Additionally, *Parabacteroides distasonis*, and a sequence that shares 99.86% identity with a *R. mucilaginosa*, emerged from this analysis as more abundant in the malaria-susceptible infants (**Supplementary Table 2**). Finally, *L. gasseri* (also identified by ANCOM-BC, **Figure 5A**) and a variant with >99.5% identity with uncultured *Veillonella* clones were elevated in the malaria-resistant infants.

## 4 Discussion

This study provides a unique look at the development of the infant gut microbiome beginning in early life, extending longitudinally over the first year of life, in a malaria-endemic region of eastern DRC. Deep sequencing of infant fecal samples, covering the entire 16S rRNA gene, revealed differences in relative abundances of a number of bacteria as a function of infant malaria susceptibility over the one year follow up period. Classifier analysis applied to the data derived from the six-week infant fecal samples showed preliminary predictive capacity for subsequent malaria risk up to one year of age, a novel and important contribution to our understanding of the relationship between the early life gut microbiome and susceptibility to malaria. The findings suggest that oral and respiratory tract bacteria (*Streptococcus, Rothia*) along with opportunistic pathogens (*Klebsiella pneumoniae, Morganella morganii*) may serve as important biomarkers for malaria risk classification in early life, while certain beneficial gut bacteria (*Bifidobacterium, Cutibacterium avidum, Megasphaera micronuciformis*) were associated with malaria resistance. The species-level k-NN model achieved 90% balanced accuracy on training set cross-validation, while repeated cross-validation and leave-one-out approaches yielded more conservative estimates of out-of-sample performance (AUC ~0.64, balanced accuracy ~62%), consistent with the small sample size and exploratory nature of the analysis. These results suggest that while the six week microbiome carries a detectable signal associated with malaria susceptibility, the predictive capacity is modest and requires validation in larger, independent cohorts before any screening application could be considered. While studies in mice and older infants and children have emphasized the relationships between the gut microbiota and malaria susceptibility and disease severity (Mandal et al., 2021; Mandal et al., 2023; Bednarski et al., 2025), the results of this study emphasize the importance of continued efforts to identify health and resistance-associated microbial elements and potentially, consortia, that might be amenable to early life interventions, such as probiotics, to protect against malaria and poor disease outcomes.

Among the early life predictors of malaria protection, *M. micronuciformis* was unexpected. It is described as an anaerobic pathogen of the oral cavity (Wang et al., 2022; Herreros-Pomares et al., 2023) and the female reproductive tract, where it induces inflammation (Salliss et al., 2021) and human papilloma virus replication (Nicolo et al., 2023) in the cervical epithelium and associates with bacterial vaginosis (Xia et al., 2016). Despite these disease associations, to our knowledge there is no evidence that this bacterium, perhaps acquired via vaginal birth, is pathogenic in the infant gut microbiome. Indeed, *M. micronuciformis* was also elevated at six weeks and three months among infants in this same cohort whose mothers’ pre-natal exposure to psychosocial stress was low, relative to those infants exposed antenatally to high stress (Dutton et al., 2023). Thus, this bacterium appears to be associated with a healthy and health-promoting gut microbiome in this population.

*Bifidobacterium* is widely considered to be an important component of an infant’s healthy gut microbiome (Dargenio et al., 2024). *B. breve* is used as a probiotic in infants to maintain and/or restore a healthy gut microbiome (Bozzi Cionci et al., 2018). Our results are consistent with other studies showing that *B. longum* (subspecies *infantis*) and *B. breve* are predominant bacteria found in the gut during early life (Jost et al., 2014; Dargenio et al., 2024). *B. breve* is acquired by the infant from maternal gut and breastmilk microbiota and potentially the vagina (Chaban et al., 2014; Jost et al., 2014; Milani et al., 2015). Colonization is supported by breastfeeding owing to its role as an efficient metabolizer of breast milk oligosaccharides (Turroni et al., 2018). Most infants in this study were exclusively breastfed through three months of age, providing a compelling basis for the early predominance of these bacteria. Despite this, lower relative abundance of this genus was consistently identified by several analytical and technical approaches, including digital PCR, among malaria-susceptible infants. These data are consistent with observations made among older children in malaria-endemic Mali and in Ugandan infants and children where increased prospective risk for any malaria infection (Yooseph et al., 2015) or for febrile malaria (Van Den Ham et al., 2024) and severe malaria (Bednarski et al., 2025) were associated with lower relative abundance of *Bifidobacterium*. Though infants in the present study did not exhibit severe malaria, our passive detection of malaria ensured that when infants appeared or behaved in a manner that suggested they were unwell, the mothers presented them for diagnosis and treatment. Thus, as a rough approximation, they can be considered to have had symptomatic malaria. Notably, among older children in Mali, higher abundance of gut *Bifidobacterium* predicted remaining malaria-free for longer than children with lower abundance, but was unrelated to the development of symptomatic malaria (Yooseph et al., 2015). The effectiveness of *B. breve* as a probiotic treatment in murine malaria, where, combined with *Lactobacillus casei* it suppressed parasitemia as well as inflammatory cytokines (Fitri et al., 2023), hints both at a potential mechanistic basis for the association of *B. breve* with resistance to malaria in infants and a potential therapeutic or preventative adjunctive measure for protection of infants in malarious areas.

The safety profile of *Bifidobacterium* and *Lactobacillus* as probiotics in term and preterm infants is well established through multiple systematic reviews and clinical trials. However, probiotic administration in resource-limited, malaria-endemic settings presents practical and regulatory challenges that would need to be addressed. It is important to emphasize that the current study establishes associations, not causation, and that the mechanism(s) by which these taxa might influence malaria susceptibility remain to be determined.

A third species identified as associated with malaria resistance at six weeks of age was *C. avidum* (formerly known as *Propionibacterium avidum*). This opportunistic bacterium may play different roles in the human host, depending on body site that is colonized (Corvec, 2018), in a strain-dependent manner (Rocha Martin et al., 2019a). It is potentially acquired from the mother through skin contact, especially through breastfeeding (Urbaniak et al., 2014), and at least one strain is specialized to inhabit the infant gut microbiome (Rocha Martin et al., 2019a). It is prevalent in the infant gut microbiome early in life, steadily decreasing with age (Rocha Martin et al., 2019b). Its association with antimalarial protection together with *B. breve* is intriguing because these two taxa are metabolically linked. *Bifidobacterium* spp. produce lactate and monosaccharides through digestion of breast milk oligosaccharides, which *C. avidum*, as a consumer of these metabolites, requires to colonize the infant gut (Rocha Martin et al., 2019a; Rocha Martin et al., 2019b). It will be important for future studies to expand on the current results with metagenomics to identify potential metabolic associations and links to malaria resistance.

Though not appearing in the six week classifier analysis, *Lactobacillus gasseri* was revealed as more abundant by DAR analysis in six-week old malaria-resistant infants, and was conspicuously persistent among these infants to at least six months of age, albeit at low abundance and low frequency. *Lactobaccillus* has long been considered an essential component of a healthy gut microbiome (Heczko et al., 2024) and is prominent in probiotic preparations intended to maintain and restore gut health (Shah et al., 2024). Lactobacilli, including *L. gasseri*, are also prominent and important functional components of the healthy vaginal microbiome (Das and Konwar, 2025). Other studies of the relationship between gut microbiota and malaria infection in infants and children have not reported an association with protection (Yooseph et al., 2015; Mandal et al., 2021; Van Den Ham et al., 2024), though in severe malaria, abundance of this genus was negatively associated with total leukocyte and neutrophil counts and heme oxygenase levels, all indicators of disease (Bednarski et al., 2025). Furthermore, *Lactobacillus* figured prominently in the gut of malaria-resistant mice, and delivery of these bacteria, along with *Bifidobacterium*, as a probiotic conferred resistance to severe malaria in this mouse model (Villarino et al., 2016). Further work to assess the importance of Lactobacilli in malaria infection is therefore warranted.

Conversely, our results align with previous reports showing associations between specific taxa and both malaria susceptibility and disease severity in children (Mandal et al., 2021; Mandal et al., 2023; Van Den Ham et al., 2024). Among Uganda children aged 0.5 to 4 years (Mandal et al., 2021), *Streptococcus salivarius* and *Klebsiella pneumoniae*, both of which were strong predictors of malaria susceptibility in six-week fecal samples in our analysis, were identified in association with severe malarial anemia. Dominance of Enterobacteriaceae, inclusive of *Klebsiella*, was recently demonstrated in young children with severe malaria and predicted mortality (Bednarski et al., 2025). Interestingly, while *Bacteroides* spp. have also been linked to susceptibility to severe malaria in young children, and multiple species function in a network to mediate severe disease in mice (Mandal et al., 2023), this genus was not discriminatory for malaria susceptibility in six-week infant samples. However, in our infants at three months of age, *B. ovatus* and *B. thetaiotaomicron* were elevated in malaria-susceptible infants whereas *B. uniformis* and *B. vulgatus* were more abundant in malaria-resistant infants (**Figure 5B**), the latter remaining elevated at 6 months as well (**Figure 5C**). These results contrast to the case of severe malarial anemia, where *B. uniformis, B. vulgatus* and *B. thetaiotaomicron* were all more abundant than in children with uncomplicated malaria (Mandal et al., 2023). Moreover, whereas *B. uniformis* and *B. ovatus* promoted malarial hyperparasitemia in mice, *B. thetaiotaomicron* reduced susceptibility (Mandal et al., 2023). The basis for this discrepancy is unclear but may be related to age differences between the two cohorts.

Aside from a compelling indication that the early infant gut microbiome can predict subsequent malaria susceptibility, the current work also indicates a dysbiotic effect of malaria infection. Other studies in children have found no or minimal impact on gut microbiota with a single or asymptomatic *P. falciparum* infection, based on V3-V4 (Mandal et al., 2019) and full variable region 16S rRNA sequencing (Bednarski et al., 2025). Severe malaria, however, was associated with significant, but rapidly resolved, dysbiosis that was associated with antibiotic use, elevated neutrophils, free hemoglobin and heme, and elevated heme oxygenase and uric acid levels (Bednarski et al., 2025). While none of the infants in the current study were diagnosed with severe malaria, they will have been symptomatic enough to prompt a visit to hospital; thus, they may represent an intermediate between the asymptomatic children and severely ill patients recently reported, among whom only the latter had profound gut dysbiosis (Bednarski et al., 2025). The factors that drive these differences remain to be fully characterized, in particular, the extent to which parasitological factors, in addition to host and extrinsic factors like nutrition and medication use (Bednarski et al., 2025) might drive dysbiosis. A role for *P. falciparum* sequestration as a driver of intestinal injury (Seydel et al., 2006; Milner et al., 2015; Church et al., 2016; Sarangam et al., 2022), inflammation and dysbiosis (Mooney et al., 2015; Taniguchi et al., 2015; Denny et al., 2019) is likely. Importantly, malaria infection is known to promote bacteremia, especially in children (Mabey et al., 1987; Scott et al., 2011; Were et al., 2011; Church and Maitland, 2014). This may explain why a significantly higher proportion of malaria-susceptible infants in our cohort had received antibiotics during the second half of the follow up period than malaria-resistant infants. It will be important for future studies examining the complex interplay between malaria infection, gut health and the gut microbiome to not only account for antibiotic use but also to assess biomarkers of gut permeability and bacteremia.

While we have identified specific taxa that predict resistance and susceptibility to malaria in this infant cohort, the mechanism(s) by which the gut microbiota might influence these outcomes remains to be established. Modulation of immune responses during early stages of neonatal immune maturation represents one possibility. A role for the gut microbiota in influencing immune development in early life, and vice versa, is well established (Zhang et al., 2022; Donald and Finlay, 2023). In general, a healthy infant gut environment helps direct the immune system toward tolerance rather than inflammation, establishing a foundation for lifelong immune balance. For example, infant-type *Bifidobacterium* species, identified in this study as associated with malaria resistance, are key early colonizers that interact with the host to promote immune development and induce anti-inflammatory gene expression to support tolerance of commensal microbes (Lin et al., 2022). Another bacterial group that helps to shape immune tolerance in infancy is *Lactobacillus*. Members of this genus play a role in steering immune development towards tolerance (McCauley et al., 2022) through suppression of inflammatory signaling (Petrof et al., 2009) in antigen-presenting cells (Villena et al., 2012) and promotion of regulatory T-cell activation (Ludwig et al., 2018). B cells in early life are also biased toward a regulatory phenotype, with rapid, but low affinity, IgM-dominated responses (Grimsholm et al., 2020; Budeus et al., 2021). Interestingly, early colonization of the infant gut with *Bifidobacterium*, as found here, and *Escherichia coli* is associated with enhanced memory B cell levels in four and eighteen month old infants (Lundell et al., 2012). This is of particular interest for anti-malaria immunity, since a role for the gut microbiome in modulating splenic anti-malarial B cell and antibody responses has been established in mouse models (Waide et al., 2020; Mandal et al., 2021). Future prospective studies that evaluate the early infant gut microbiome with immune development, in particular development of anti-malarial humoral immunity, are needed to identify if such an association occurs in humans.

Among the earliest colonizers of the infant gut are *Enterobacteriaceae*, including *Escherichia*, *Enterobacter*, and *Klebsiella*, which play a temporary but important role in shaping the infant gut environment by consuming oxygen and facilitating the transition to an anaerobic environment that support strict anaerobes such as *Bifidobacterium* and *Lactobacillus* (Arrieta et al., 2014; Moreira de Gouveia et al., 2024). Sustained colonization by *Enterobacteriaceae*, however, co-occurring with delayed colonization of *Bifidobacterium*, has been linked to intestinal inflammation and risk of allergic and metabolic disease later in life (Rey-Marino and Francino, 2022). It is intriguing that we observed dominance of *Klebsiella* as a key indicator of malaria susceptibility in the six-week infant gut microbiome, and that severe malaria was characterized by elevated abundance of *Enterobacteriaceae* in infants and young children, aged six months to four years, who were susceptible to severe malaria and poor disease outcomes (Bednarski et al., 2025).

Several limitations of the current study restrict our conclusions. The cohort of 47 infants, while comparable to other studies in this field (Bednarski et al., 2025; Xue et al., 2025), limits statistical power to detect small effect sizes; *post-hoc* analysis indicates that only large effects (Cohen’s d ≥ 0.89) are detectable at 80% power. The 75/25 train/test split yielded a test set of only 12 samples, and repeated cross-validation, LOOCV, and permutation testing indicate that out-of-sample predictive capacity is modest (AUC ~0.64, balanced accuracy ~62%; permutation p = 0.312). Bednet use at six weeks was a significant confounder that improved classifier performance when included. Additional limitations include lack of information on maternal microbiota, limited follow-up sampling points, infrequent and passive detection of malaria cases, and the inherent limitation of 16S rRNA sequencing, which does not provide functional, metabolic, or true strain-level information. The associations described between specific taxa and malaria outcomes are correlative and do not demonstrate causation. Metagenomic, metatranscriptomic, and metabolomic follow-up studies will be necessary to characterize the functional capacity of the implicated taxa. Follow up studies examining the factors influencing the earliest stages of infant gut microbiome establishment in the context of malaria risk and how these equate to immune development in infancy, particularly as it relates to antimalarial immunity, will be important. Notably, ANCOM-BC differential abundance results at six weeks were robust to bednet adjustment, with 11 taxa retaining significance (q < 0.05). Should follow-up studies, ideally in larger and independent cohorts, confirm and extend the microbial associations identified here, and establish causal mechanisms through metagenomic and functional analyses, investigation of microbiome-targeted interventions for malaria prevention in early life may be warranted. However, given the preliminary nature of the current predictive findings and the observational design of this study, substantial additional work, including mechanistic studies and assessment of probiotic safety and efficacy in this population, would be required before any interventional approach could be justified.

## Data Availability

The datasets presented in this study can be found in online repositories. Raw 16S rRNA sequencing data are available in the NCBI BioProject database under accession PRJNA903474. Analysis code, intermediate data products, and the full reproducible workflow are archived on Zenodo (DOI: 10.5281/zenodo.19634437) and maintained at https://github.com/UFDuttonLab/congo-malaria-microbiome.

## References

[B1] Arrieta M. C. Stiemsma L. T. Amenyogbe N. Brown E. M. Finlay B. (2014). The intestinal microbiome in early life: health and disease. Front. Immunol. 5, 427. doi: 10.3389/fimmu.2014.00427 25250028 PMC4155789

[B2] Ashton P. M. Mageiros L. Meiring J. E. Chunga-Chirambo A. Khanam F. Dongol S. . (2025). Interplay between the gut microbiome and typhoid fever: Insights from endemic countries and a controlled human infection model. Microbiome 13, 168. doi: 10.1186/s40168-025-02125-7 40696437 PMC12281769

[B3] Bednarski O. J. Lehman S. B. Mzinza D. Kazinga C. Namazzi R. Opoka R. O. . (2025). Gut bacterial dysbiosis in pediatric severe malaria associates with post-discharge mortality. Nat. Commun. 16, 9658. doi: 10.1038/s41467-025-64632-3 41173910 PMC12578976

[B4] Bozzi Cionci N. Baffoni L. Gaggia F. Di Gioia D. (2018). Therapeutic microbiology: The role of Bifidobacterium breve as food supplement for the prevention/treatment of pediatric diseases. Nutrients 10 (11), 1723. doi: 10.3390/nu10111723 30423810 PMC6265827

[B5] Budeus B. Kibler A. Brauser M. Homp E. Bronischewski K. Ross J. A. . (2021). Human cord blood B cells differ from the adult counterpart by conserved Ig repertoires and accelerated response dynamics. J. Immunol. 206, 2839–2851. doi: 10.4049/jimmunol.2100113 34117106

[B6] Chaban B. Links M. G. Jayaprakash T. P. Wagner E. C. Bourque D. K. Lohn Z. . (2014). Characterization of the vaginal microbiota of healthy Canadian women through the menstrual cycle. Microbiome 2, 23. doi: 10.1186/2049-2618-2-23 25053998 PMC4106219

[B7] Chua C. L. L. Hasang W. Rogerson S. J. Teo A. (2021). Poor birth outcomes in malaria in pregnancy: Recent insights into mechanisms and prevention approaches. Front. Immunol. 12, 621382. doi: 10.3389/fimmu.2021.621382 33790894 PMC8005559

[B8] Church J. Maitland K. (2014). Invasive bacterial co-infection in African children with Plasmodium falciparum malaria: a systematic review. BMC Med. 12, 31. doi: 10.1186/1741-7015-12-31 24548672 PMC3928319

[B9] Church J. A. Nyamako L. Olupot-Olupot P. Maitland K. Urban B. C. (2016). Increased adhesion of Plasmodium falciparum infected erythrocytes to ICAM-1 in children with acute intestinal injury. Malar J. 15, 54. doi: 10.1186/s12936-016-1110-3 26830671 PMC4736236

[B10] Corvec S. (2018). Clinical and biological features of Cutibacterium (formerly Propionibacterium) avidum, an underrecognized microorganism. Clin. Microbiol. Rev. 31. doi: 10.1128/cmr.00064-17 29848774 PMC6056840

[B11] Dargenio V. N. Cristofori F. Brindicci V. F. Schettini F. Dargenio C. Castellaneta S. P. . (2024). Impact of Bifidobacterium longum subspecies infantis on pediatric gut health and nutrition: Current evidence and future directions. Nutrients 16 (20), 3510. doi: 10.3390/nu16203510 39458503 PMC11510697

[B12] Das S. Konwar B. K. (2025). The vital role of Lactobacillus sp. in vaginal health: Implications for enhanced prophylactic research. Probio Antimicrob. Proteins. 18 (1), 1602–1621. doi: 10.1007/s12602-025-10574-7 40402415

[B13] Denny J. E. Powell W. L. Schmidt N. W. (2016). Local and long-distance calling: Conversations between the gut microbiota and intra- and extra-gastrointestinal tract infections. Front. Cell. Infect. Microbiol. 6, 41. doi: 10.3389/fcimb.2016.00041 27148490 PMC4826874

[B14] Denny J. E. Powers J. B. Castro H. F. Zhang J. Joshi-Barve S. Campagna S. R. . (2019). Differential sensitivity to Plasmodium yoelii infection in C57BL/6 mice impacts gut-liver axis homeostasis. Sci. Rep. 9, 3472. doi: 10.1038/s41598-019-40266-6 30837607 PMC6401097

[B15] Desai M. Ter Kuile F. O. Nosten F. McGready R. ASamoa K. Brabin B. . (2007). Epidemiology and burden of malaria in pregnancy. Lancet Infect. Dis. 7, 93–104. doi: 10.1016/s1473-3099(07)70021-x 17251080

[B16] Donald K. Finlay B. B. (2023). Early-life interactions between the microbiota and immune system: impact on immune system development and atopic disease. Nat. Rev. Immunol. 23, 735–748. doi: 10.1038/s41577-023-00874-w 37138015

[B17] Dutton C. L. Maisha F. M. Quinn E. B. Morales K. L. Moore J. M. Mulligan C. J. (2023). Maternal psychosocial stress is associated with reduced diversity in the early infant gut microbiome. Microorganisms 11 (4), 975. doi: 10.3390/microorganisms11040975 37110398 PMC10142543

[B18] Ferretti P. Pasolli E. Tett A. Asnicar F. Gorfer V. Fedi S. . (2018). Mother-to-infant microbial transmission from different body sites shapes the developing infant gut microbiome. Cell Host Microbe 24, 133–145. doi: 10.1016/j.chom.2018.06.005 30001516 PMC6716579

[B19] Fitri L. E. Sardjono T. W. Winaris N. Pawestri A. R. Endharti A. T. Norahmawati E. . (2023). Bifidobacterium longum administration diminishes parasitemia and inflammation during Plasmodium berghei infection in mice. J. Inflamm. Res. 16, 1393–1404. doi: 10.2147/jir.s400782 37006809 PMC10065020

[B20] Grimsholm O. Piano Mortari E. Davydov A. N. Shugay M. Obraztsova A. S. Bocci C. . (2020). The interplay between CD27(dull) and CD27(bright) B cells ensures the flexibility, stability, and resilience of human B cell memory. Cell Rep. 30, 2963–2977. doi: 10.1016/j.celrep.2020.02.022 32130900

[B21] Hartman T. K. Rogerson S. J. Fischer P. R. (2010). The impact of maternal malaria on newborns. Ann. Trop. Paediatr. 30, 271–282. doi: 10.1179/146532810x12858955921032 21118620

[B22] Heczko P. B. Giemza M. Ponikiewska W. Strus M. (2024). Importance of Lactobacilli for human health. Microorganisms 12 (12), 2382. doi: 10.3390/microorganisms12122382 39770585 PMC11676770

[B23] Herreros-Pomares A. Hervas D. Bagan-Debon L. Jantus-Lewintre E. Gimeno-Cardona C. Bagan J. (2023). On the oral microbiome of oral potentially Malignant and Malignant disorders: Dysbiosis, loss of diversity, and pathogens enrichment. Int. J. Mol. Sci. 24 (4), 3466. doi: 10.3390/ijms24043466 36834903 PMC9961214

[B24] Jost T. Lacroix C. Braegger C. P. Rochat F. Chassard C. (2014). Vertical mother-neonate transfer of maternal gut bacteria via breastfeeding. Environ. Microbiol. 16, 2891–2904. doi: 10.1111/1462-2920.12238 24033881

[B25] Lin C. Lin Y. Zhang H. Wang G. Zhao J. Zhang H. . (2022). Intestinal 'Infant-Type' Bifidobacteria mediate immune system development in the first 1000 days of life. Nutrients 14 (7), 1498. doi: 10.3390/nu14071498 35406110 PMC9002861

[B26] Lin H. Peddada S. D. (2020). Analysis of compositions of microbiomes with bias correction. Nat. Commun. 11, 3514. doi: 10.1038/s41467-020-17041-7 32665548 PMC7360769

[B27] Liu C. Mansoldo F. R. P. Li H. Vermelho A. B. Zeng R. J. Li X. . (2025). A workflow for statistical analysis and visualization of microbiome omics data using the R microeco package. Nat. Protoc. 21 (4), 1300–1324. doi: 10.1038/s41596-025-01239-4 40770112

[B28] Ludwig I. S. Broere F. Manurung S. Lambers T. T. van der Zee R. van Eden W. (2018). Lactobacillus rhamnosus GG-derived soluble mediators modulate adaptive immune cells. Front. Immunol. 9, 1546. doi: 10.3389/fimmu.2018.01546 30042761 PMC6048560

[B29] Lundell A. C. Bjornsson V. Ljung A. Ceder M. Johansen S. Lindhagen G. . (2012). Infant B cell memory differentiation and early gut bacterial colonization. J. Immunol. 188, 4315–4322. doi: 10.4049/jimmunol.1103223 22490441

[B30] Mabey D. C. Brown A. Greenwood B. M. (1987). Plasmodium falciparum malaria and Salmonella infections in Gambian children. J. Infect. Dis. 155, 1319–1321. doi: 10.1093/infdis/155.6.1319 3553352

[B31] Mandal R. K. Crane R. J. Berkley J. A. Gumbi W. Wambua J. Ngoi J. M. . (2019). Longitudinal analysis of infant stool bacteria communities before and after acute febrile malaria and artemether-lumefantrine treatment. J. Infect. Dis. 220, 687–698. doi: 10.1093/infdis/jiy740 30590681 PMC6639600

[B32] Mandal R. K. Denny J. E. Namazzi R. Opoka R. O. Datta D. John C. C. . (2021). Dynamic modulation of spleen germinal center reactions by gut bacteria during Plasmodium infection. Cell Rep. 35, 109094. doi: 10.1016/j.celrep.2021.109094 33979614 PMC8141963

[B33] Mandal R. K. Mandal A. Denny J. E. Namazii R. John C. C. Schmidt N. W. (2023). Gut Bacteroides act in a microbial consortium to cause susceptibility to severe malaria. Nat. Commun. 14, 6465. doi: 10.1038/s41467-023-42235-0 37833304 PMC10575898

[B34] Mandal R. K. Schmidt N. W. (2023). Mechanistic insights into the interaction between the host gut microbiome and malaria. PloS Pathog. 19, e1011665. doi: 10.1371/journal.ppat.1011665 37824458 PMC10569623

[B35] Mathew S. Smatti M. K. Al Ansari K. Nasrallah G. K. Al Thani A. A. Yassine H. M. (2019). Mixed viral-bacterial infections and their effects on gut microbiota and clinical illnesses in children. Sci. Rep. 9, 865. doi: 10.1038/s41598-018-37162-w 30696865 PMC6351549

[B36] McCartney A. L. Hoyles L. (2023). The role of Klebsiella populations in preterm infants. Biochem. Soc Trans. 51, 887–896. doi: 10.1042/bst20200325 37099394 PMC10212511

[B37] McCauley K. E. Rackaityte E. LaMere B. Fadrosh D. W. Fujimura K. E. Panzer A. R. . (2022). Heritable vaginal bacteria influence immune tolerance and relate to early-life markers of allergic sensitization in infancy. Cell Rep. Med. 3, 100713. doi: 10.1016/j.xcrm.2022.100713 35932762 PMC9418802

[B38] McMurdie P. J. Holmes S. (2013). phyloseq: An R package for reproducible interactive analysis and graphics of microbiome census data. PloS One 8, e61217. doi: 10.1371/journal.pone.0061217 23630581 PMC3632530

[B39] Milani C. Mancabelli L. Lugli G. A. Duranti S. Turroni F. Ferrario C. . (2015). Exploring vertical transmission of Bifidobacteria from mother to child. Appl. Environ. Microbiol. 81, 7078–7087. doi: 10.1128/aem.02037-15 26231653 PMC4579462

[B40] Milner D. A. Lee J. J. Frantzreb C. Whitten R. O. Kamiza S. Carr R. A. . (2015). Quantitative assessment of multiorgan sequestration of parasites in fatal pediatric cerebral malaria. J. Infect. Dis. 212(8), 1317–1321. doi: 10.1093/infdis/jiv205 25852120 PMC4577044

[B41] Mooney J. P. Lokken K. L. Byndloss M. X. George M. D. Velazquez E. M. Faber F. . (2015). Inflammation-associated alterations to the intestinal microbiota reduce colonization resistance against non-typhoidal Salmonella during concurrent malaria parasite infection. Sci. Rep. 5, 14603. doi: 10.1038/srep14603 26434367 PMC4592952

[B42] Moore J. M. Morales Aparicio J. C. (2022). Enhancing pathogen resistance: The gut microbiota and malaria. Ed. Glibetic M. Comprehensive Gut Microbiota ( Elsevier), 143–167. doi: 10.1016/B978-0-12-819265-8.00097-8

[B43] Moreira de Gouveia M. I. Bernalier-Donadille A. Jubelin G. (2024). Enterobacteriaceae in the human gut: dynamics and ecological roles in health and disease. Biol. Bsl 13 (3), 142. doi: 10.3390/biology13030142 38534413 PMC10967970

[B44] Morffy Smith C. D. Gong M. Andrew A. K. Russ B. N. Ge Y. Zadeh M. . (2019). Composition of the gut microbiota transcends genetic determinants of malaria infection severity and influences pregnancy outcome. EBioMedicine 44, 639–655. doi: 10.1016/j.ebiom.2019.05.052 31160271 PMC6606560

[B45] Muller O. Traore C. Kouyate B. Ye Y. Frey C. Coulibaly B. . (2006). Effects of insecticide-treated bednets during early infancy in an African area of intense malaria transmission: A randomized controlled trial. Bull. World Health Organ 84, 120–126. doi: 10.2471/blt.05.023150 16501729 PMC2626534

[B46] Nicolo S. Antonelli A. Tanturli M. Baccani I. Bonaiuto C. Castronovo G. . (2023). Bacterial species from vaginal microbiota differently affect the production of the E6 and E7 oncoproteins and of p53 and p-Rb oncosuppressors in HPV16-infected cells. Int. J. Mol. Sci. 24 (8), 7173. doi: 10.3390/ijms24087173 37108333 PMC10138431

[B47] Olm M. R. Bhattacharya N. Crits-Christoph A. Firek B. A. Baker R. Song Y. S. . (2019). Necrotizing enterocolitis is preceded by increased gut bacterial replication, Klebsiella, and fimbriae-encoding bacteria. Sci. Adv. 5, eaax5727. doi: 10.1126/sciadv.aax5727 31844663 PMC6905865

[B48] Petrof E. O. Claud E. C. Sun J. Abramova T. Guo Y. Waypa T. S. . (2009). Bacteria-free solution derived from Lactobacillus plantarum inhibits multiple NF-kappaB pathways and inhibits proteasome function. Inflamm. Bowel Dis. 15, 1537–1547. doi: 10.1002/ibd.20930 19373789 PMC2748164

[B49] Rey-Marino A. Francino M. P. (2022). Nutrition, gut microbiota, and allergy development in infants. Nutrients 14 (20), 4316. doi: 10.3390/nu14204316 36297000 PMC9609088

[B50] Rocha Martin V. N. Lacroix C. Killer J. Bunesova V. Voney E. Braegger C. . (2019a). Cutibacterium avidum is phylogenetically diverse with a subpopulation being adapted to the infant gut. Syst. Appl. Microbiol. 42, 506–516. doi: 10.1016/j.syapm.2019.05.001 31128887

[B51] Rocha Martin V. N. Schwab C. Krych L. Voney E. Geirnaert A. Braegger C. . (2019b). Colonization of Cutibacterium avidum during infant gut microbiota establishment. FEMS Microbiol. Ecol. 95 (4), 506–516. doi: 10.1097/mco.0000000000000463 30388209

[B52] Salliss M. E. Maarsingh J. D. Garza C. Laniewski P. Herbst-Kralovetz M. M. (2021). Veillonellaceae family members uniquely alter the cervical metabolic microenvironment in a human three-dimensional epithelial model. NPJ Biofilms Microbiomes 7, 57. doi: 10.1038/s41522-021-00229-0 34230496 PMC8260719

[B53] Sarangam M. L. Namazzi R. Datta D. Bond C. Vanderpool C. P. B. Opoka R. O. . (2022). Intestinal injury biomarkers predict mortality in pediatric severe malaria. mBio 13, e0132522. doi: 10.1128/mbio.01325-22 36069443 PMC9601216

[B54] Sarker S. A. Sultana S. Reuteler G. Moine D. Descombes P. Charton F. . (2016). Oral phage therapy of acute bacterial diarrhea with two coliphage preparations: A randomized trial in children from Bangladesh. EBioMedicine 4, 124–137. doi: 10.1016/j.ebiom.2015.12.023 26981577 PMC4776075

[B55] Scott J. A. Berkley J. A. Mwangi I. Ochola L. Uyoga S. Macharia A. . (2011). Relation between falciparum malaria and bacteremia in Kenyan children: a population-based, case-control study and a longitudinal study. Lancet 378, 1316–1323. doi: 10.1016/s0140-6736(11)60888-x 21903251 PMC3192903

[B56] Seydel K. B. Milner D. A. Kamiza S. B. Molyneux M. E. Taylor T. E. (2006). The distribution and intensity of parasite sequestration in comatose Malawian children. J. Infect. Dis. 194, 208–205. doi: 10.1086/505078 16779727 PMC1515074

[B57] Shah A. B. Baiseitova A. Zahoor M. Ahmad I. Ikram M. Bakhsh A. . (2024). Probiotic significance of Lactobacillus strains: a comprehensive review on health impacts, research gaps, and future prospects. Gut Microbes 16, 2431643. doi: 10.1080/19490976.2024.2431643 39582101 PMC11591481

[B58] Shields-Cutler R. R. Al-Ghalith G. A. Yassour M. Knights D. (2018). SplinectomeR enables group comparisons in longitudinal microbiome studies. Front. Microbiol. 9, 785. doi: 10.3389/fmicb.2018.00785 29740416 PMC5924793

[B59] Stewart C. J. Ajami N. J. O'Brien J. L. Hutchinson D. S. Smith D. P. Wong M. C. . (2018). Temporal development of the gut microbiome in early childhood from the TEDDY study. Nature 562, 583–588. doi: 10.1038/s41586-018-0617-x 30356187 PMC6415775

[B60] Taniguchi T. Miyauchi E. Nakamura S. Hirai M. Suzue K. Imai T. . (2015). Plasmodium berghei ANKA causes intestinal malaria associated with dysbiosis. Sci. Rep. 5, 15699. doi: 10.1038/srep15699 26503461 PMC4621605

[B61] Turroni F. Milani C. Duranti S. Ferrario C. Lugli G. A. Mancabelli L. . (2018). Bifidobacteria and the infant gut: An example of co-evolution and natural selection. Cell. Mol. Life Sci. 75, 103–118. doi: 10.1007/s00018-017-2672-0 28983638 PMC11105234

[B62] Urbaniak C. Cummins J. Brackstone M. Macklaim J. M. Gloor G. B. Baban C. K. . (2014). Microbiota of human breast tissue. Appl. Environ. Microbiol. 80, 3007–3014. doi: 10.1128/aem.00242-14 24610844 PMC4018903

[B63] Van Den Ham K. M. Bower L. K. Li S. Lorenzi H. Doumbo S. Doumtabe D. . (2024). The gut microbiome is associated with susceptibility to febrile malaria in Malian children. Nat. Commun. 15, 9525. doi: 10.1038/s41467-024-52953-8 39500866 PMC11538534

[B64] Villarino N. F. LeCleir G. R. Denny J. E. Dearth S. P. Harding C. L. Sloan S. S. . (2016). Composition of the gut microbiota modulates the severity of malaria. Proc. Natl. Acad. Sci. U.S.A. 113, 2235–2240. doi: 10.1073/pnas.1504887113 26858424 PMC4776451

[B65] Villena J. Suzuki R. Fujie H. Chiba E. Takahashi T. Tomosada Y. . (2012). Immunobiotic Lactobacillus jensenii modulates the Toll-like receptor 4-induced inflammatory response via negative regulation in porcine antigen-presenting cells. Clin. Vaccine Immunol. 19, 1038–1053. doi: 10.1128/cvi.00199-12 22573738 PMC3393362

[B66] Waide M. L. Polidoro R. Powell W. L. Denny J. E. Kos J. Tieri D. A. . (2020). Gut microbiota composition modulates the magnitude and quality of germinal centers during Plasmodium infections. Cell Rep. 33, 108503. doi: 10.1016/j.celrep.2020.108503 33326773 PMC7772993

[B67] Waldman A. J. Balskus E. P. (2018). The human microbiota, infectious disease, and global health: Challenges and opportunities. ACS Infect. Dis. 4, 14–26. doi: 10.1021/acsinfecdis.7b00232 29207239

[B68] Wang X. Luo N. Mi Q. Kong W. Zhang W. Li X. . (2022). Influence of cigarette smoking on oral microbiota in patients with recurrent aphthous stomatitis. J. Invest. Med: Off. Publ. Am. Fed. For. Clin. Res. 70, 805–813. doi: 10.1136/jim-2021-002119 34824153

[B69] Were T. Davenport G. C. Hittner J. B. Ouma C. Vulule J. M. Ong'echa J. M. . (2011). Bacteremia in Kenyan children presenting with malaria. J. Clin. Microbiol. 49, 671–676. doi: 10.1128/jcm.01864-10 21106789 PMC3043473

[B70] World Health Organization . (2024). World malaria report 2024. (Geneva: World Health Organization).

[B71] World Health Organization . (2025). World malaria report 2025: addressing the threat of antimalarial drug resistance (Geneva: World Health Organization).

[B72] Xia Q. Cheng L. Zhang H. Sun S. Liu F. Li H. . (2016). Identification of vaginal bacteria diversity and it's association with clinically diagnosed bacterial vaginosis by denaturing gradient gel electrophoresis and correspondence analysis. Infect Genet. Evol: J. Mol. Epidemiol. Evol Genet. Infect. Dis. 44, 479–486. doi: 10.1016/j.meegid.2016.08.001 27503595

[B73] Xue L. Wang C. Liu C. (2025). Immunomodulatory effects of gut microbiota on vaccine efficacy against respiratory pathogens. Front. Immunol. 16, 1618921. doi: 10.3389/fimmu.2025.1618921 40529354 PMC12170667

[B74] Yilmaz B. Portugal S. Tran T. M. Gozzelino R. Ramos S. Gomes J. . (2014). Gut microbiota elicits a protective immune response against malaria transmission. Cell. 159, 1277–1289. doi: 10.1016/j.cell.2014.10.053 25480293 PMC4261137

[B75] Yooseph S. Kirkness E. F. Tran T. M. Harkins D. M. Jones M. B. Torralba M. G. . (2015). Stool microbiota composition is associated with the prospective risk of Plasmodium falciparum infection. BMC Genomics 16, 631. doi: 10.1186/s12864-015-1819-3 26296559 PMC4546150

[B76] Zhang H. Zhang Z. Liao Y. Zhang W. Tang D. (2022). The complex link and disease between the gut microbiome and the immune system in infants. Front. Cell. Infect. Microbiol. 12, 924119. doi: 10.3389/fcimb.2022.924119 35782111 PMC9241338

