# Supplementary material for: The gut microbiome in early life predicts malaria susceptibility
Source: Front Cell Infect Microbiol. 2026 Jun 23;16:1769376. doi: 10.3389/fcimb.2026.1769376 (PMC13337902; doi:10.3389/fcimb.2026.1769376)
Supplement: Supplementary file 1 [file Table1.pdf]

Supplementary Figures

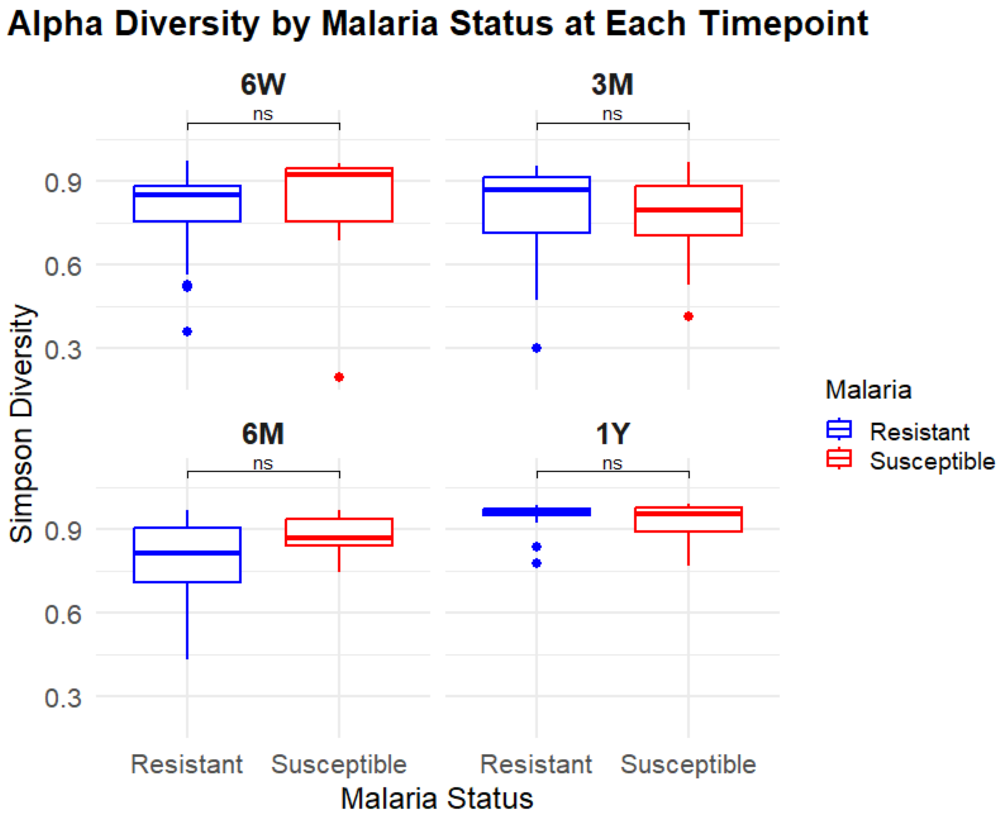

Supplementary Figure 1. Alpha diversity plots of malaria resistant versus malaria-susceptible infants at six weeks (6W), three months (3M), six months (6M) and one year of age (1Y). Plots of Simpson Diversity. All nonsignificant by Kruskal-Wallis Rank Sum Test, adjusted $p > 0.05$.

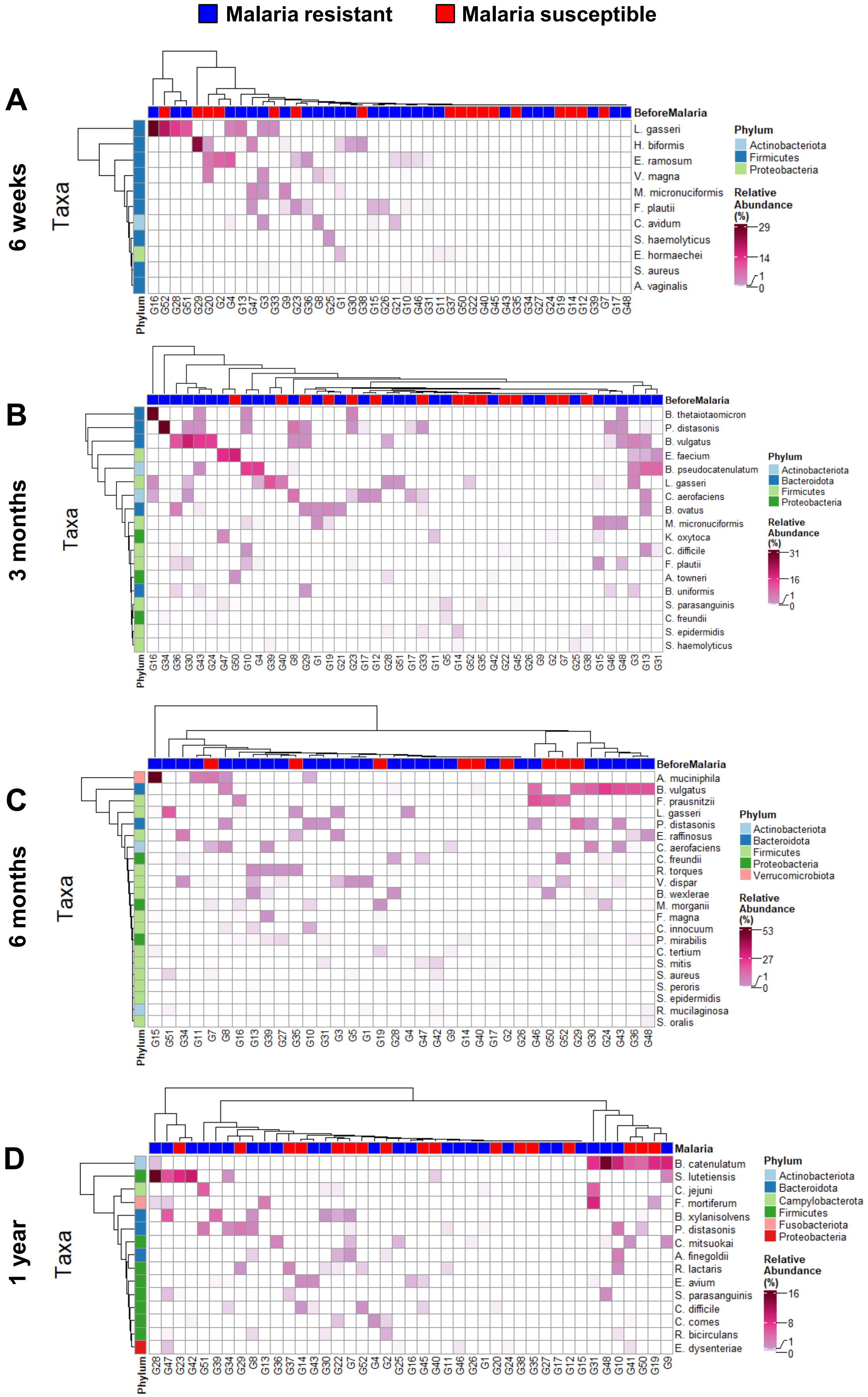
Malaria resistant
Malaria susceptible
A
6 weeks
Taxa
BeforeMalaria
L. gasseri
H. biformis
E. ramosum
V. magna
M. micronuciformis
F. plautii
C. avidum
S. haemolyticus
E. hormaechei
S. aureus
A. vaginalis
Phylum
Phylum
Actinobacteriota
Firmicutes
Proteobacteria
Relative Abundance (%)
29
14
1
0
B
3 months
Taxa
BeforeMalaria
B. thetaiotaomicron
P. distasonis
B. vulgatus
E. faecium
B. pseudocatenulatum
L. gasseri
C. aerofaciens
B. ovatus
M. micronuciformis
K. oxytoca
C. difficile
F. plautii
A. towneri
B. uniformis
S. parasanguinis
C. freundii
S. epidermidis
S. haemolyticus
Phylum
Phylum
Actinobacteriota
Bacteroidota
Firmicutes
Proteobacteria
Relative Abundance (%)
31
16
1
0
C
6 months
Taxa
BeforeMalaria
A. muciniphila
B. vulgatus
F. prausnitzii
L. gasseri
P. distasonis
E. raffinosus
C. aerofaciens
C. freundii
R. torques
V. dispar
B. wexlerae
M. morganii
F. magna
C. innocuum
P. mirabilis
C. tertium
S. mitis
S. aureus
S. peroris
S. epidermidis
R. mucilaginosa
S. oralis
Phylum
Phylum
Actinobacteriota
Bacteroidota
Firmicutes
Proteobacteria
Verrucomicrobiota
Relative Abundance (%)
53
27
1
0
D
1 year
Taxa
Malaria
B. catenulatum
S. lutetiensis
C. jejuni
F. mortiferum
B. xylanisolvens
P. distasonis
C. mitsuokai
A. finegoldii
R. lactaris
E. avium
S. parasanguinis
C. difficile
C. comes
R. bicirculans
E. dysenteriae
Phylum
Phylum
Actinobacteriota
Bacteroidota
Campylobacterota
Firmicutes
Fusobacteriota
Proteobacteria
Relative Abundance (%)
16
8
1
0

Supplementary Figure 2. Heat maps for ANCOM-BC analyses at four follow up timepoints. (A-C) Blue-headed columns represent infants who never experienced a malaria infection (malaria resistant) and red-headed columns represent all infants who, at a later timepoint, were diagnosed with malaria, at six week, three month and six month timepoints (malaria susceptible). (D) The one year time point. Blue-headed columns represent malaria-resistant infants and red-headed columns represent all infants who at some time during the one year follow up were diagnosed with malaria.

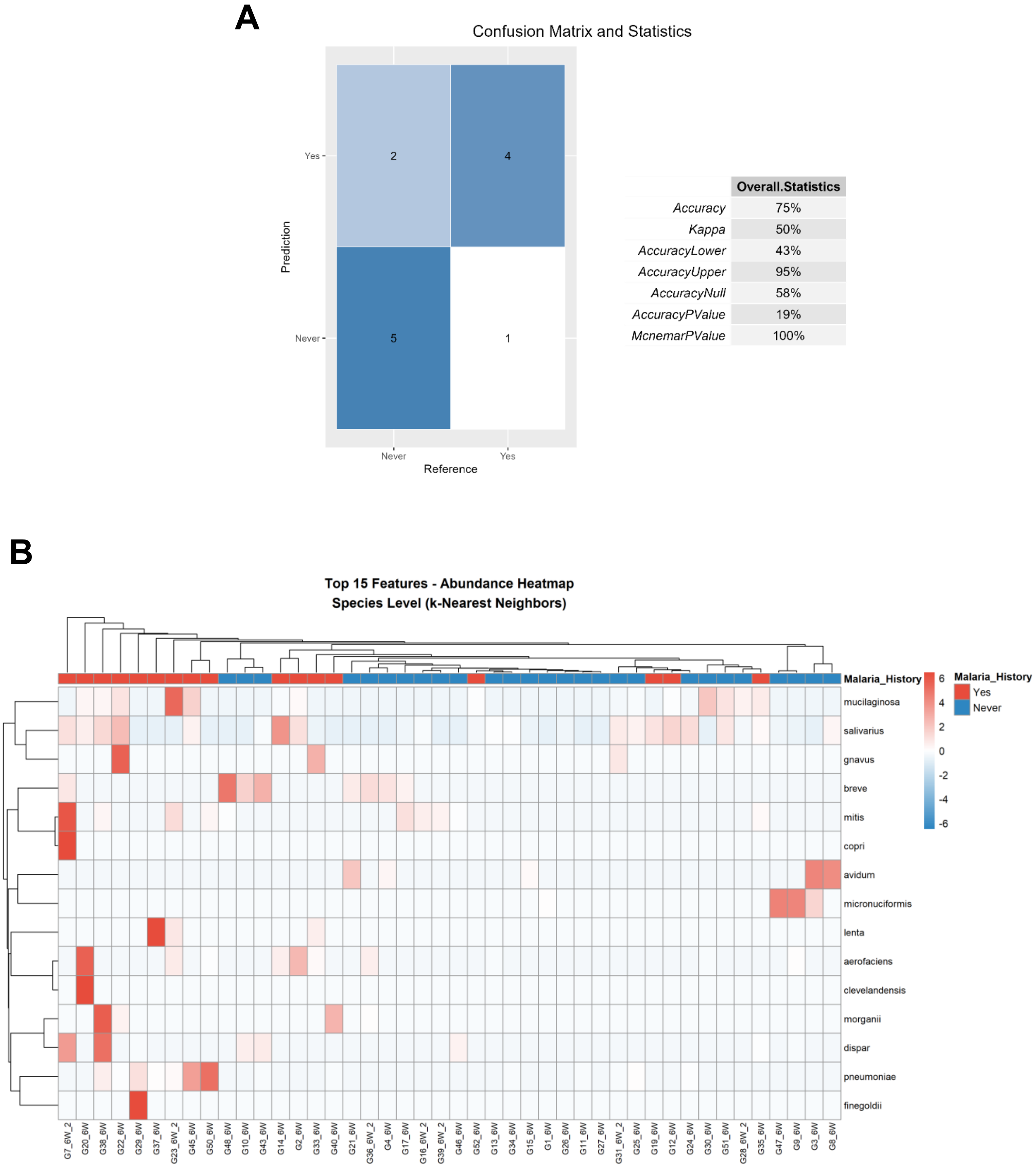

Supplementary Figure 3. k-nearest neighbors classifier analysis with Boruta selection. (A) Confusion matrix. **(B)** Abundance heatmap. “Never” refers to infants that did not have a diagnosed malaria episode (“resistant”) and “yes” refers to malaria-susceptible infants.

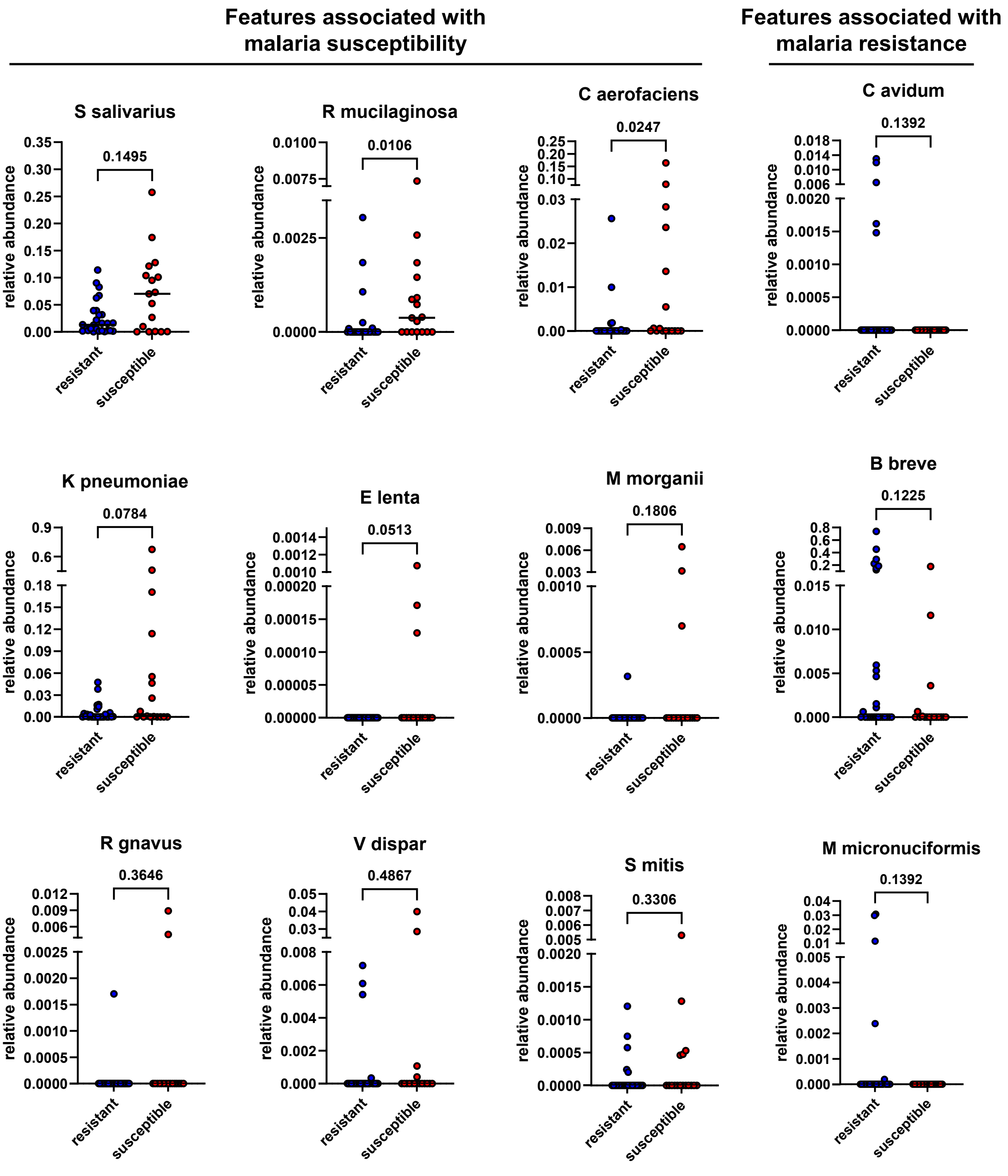

Supplementary Figure 4. Differential abundance analysis of top 12 species identified by classifier analysis in six week fecal microbiota in malaria-resistant and malaria-susceptible infants. Relative abundance at the species level in malaria-resistant infant fecal samples are shown in blue and malaria susceptible samples are shown in red. Comparisons done using Mann Whitney test.
