# Supplementary material for: The gut microbiome in early life predicts malaria susceptibility
Source: Front Cell Infect Microbiol. 2026 Jun 23;16:1769376. doi: 10.3389/fcimb.2026.1769376 (PMC13337902; doi:10.3389/fcimb.2026.1769376)
Supplement: Supplementary file 2 [file Table2.pdf]

Supplemental Table 1. Summary of Differential Abundance Analysis by Consensus (DAR) at the genus level in malaria-resistant versus malaria-susceptible infants at six weeks of age.

**Test**	**Genus**	**Effect**	**P value, adjusted**
corncob	*Bifidobacterium**	-1.63524	0.008133
DeSeq2	*Lactobacillus**	-26.2298	1.71E-14
	*Megasphaera**	-22.3131	8.38E-11
	Prevotella_9	-22.2577	8.38E-11
	*Lacticaseibacillus**	-21.1174	7.45E-10
metagenomeseq	*Intestinimonas*	10.53932	6.72E-09
	Fusicatenibacter	10.02379	3.93E-09
	*Lactobacillus**	-9.84426	1.72E-38
	unknown	8.027709	1.59E-07
	*Megasphaera**	-7.80144	8.87E-10
	Actinomyces	7.298027	2.95E-08
	[Ruminococcus] gnavus group	7.169517	1.44E-07
	Limosilactobacillus	-6.17427	2.63E-16
	Cutibacterium	-5.72776	0.000322
	Lachnoclostridium	5.279214	4.95E-09
	Morganella	-5.11064	2.42E-08
	Aerococcus	-4.60917	0.020945
	Haemophilus	4.426461	0.000138
	Citrobacter	4.282804	0.001379
	Ligilactobacillus	-3.97042	0.014316
	Collinsella	3.925238	5.03E-11
	Corynebacterium	-3.86774	0.000204
	Parabacteroides	3.220792	1.85E-08
	*Bifidobacterium**	-2.93588	1.89E-11
	Atopobium	-2.88289	0.00217
	Sutterella	2.719223	1.62E-05
	*Lacticaseibacillus**	-2.70073	0.026848
	Peptoniphilus	-2.0166	0.010754
	Staphylococcus	-1.26888	0.020945
	*Streptococcus**	1.001688	0.032918
Wilcoxon	*Bifidobacterium**	-0.31758	0.00391
	*Streptococcus**	0.066852	0.0196
	*Lactobacillus**	0	0.0455
	Rothia	0.0002	0.0468
	Klebsiella	0.010317	0.0297

*Genera identified by ≥2 tests as significant

Supplemental Table 2. Summary of Differential Abundance Analysis by Consensus (DAR) at the species level in malaria-resistant versus malaria-susceptible infants at six weeks of age.

Test	Species	Effect	P value, adjusted
DeSeq2	*L. gasseri**	-26.2085	1.75E-14
	unknown1*	-23.4544	3.63E-19
	F. plautii	-22.4974	1.68E-13
	C. butyricum	-22.1558	1.52E-10
metagenomeseq	*L. gasseri**	-11.1381	5.89E-36
	B. dentium	-8.78776	2.99E-07
	unknown	-7.17062	4.80E-05
	B. stercoris	-6.86857	0.000123
	K. oxytoca	-6.45126	6.63E-05
	unknown1*	-6.29465	6.47E-14
	C. aurimucosum	-5.97502	0.027849
	unknown	-5.74123	0.000293
	S. wadsworthensis	-5.41515	0.006909
	unknown	-5.26611	1.13E-12
	unknown	-5.2408	0.0042
	unknown	-5.20066	0.005048
	B. caccae	-4.97863	0.035288
	B. obeum	-4.91121	0.003448
	unknown	-4.80846	0.009167
	unknown	-4.66744	0.012937
	M. micronuciformis	-4.63518	0.006909
	unknown	-4.57796	5.43E-05
	S. flavescens	-4.36927	0.001416
	A. hadrus	-4.0778	0.002036
	D. invisus	-3.94463	0.038386
	unknown	-3.91487	0.041257
	unknown	-3.77182	0.035288
	C. mangenotii	-3.70365	0.027849
	unknown	-3.6536	0.009311
	A. urinaeequi	-3.6286	0.004184
	P. prevotii	-3.6105	0.005055
	C. mitsuokai	-3.57975	0.027849
	P. grossensis	-3.49472	0.028253
	C. avidum	-3.49214	0.007695
	K. quasipneumoniae	-3.38802	0.01907
	S. haemolyticus	-3.33846	9.52E-05
	B. longum	-3.11739	1.38E-12
	unknown	-2.76104	0.01907
	P. mirabilis	-2.64995	0.009167
	C. amycolatum	-2.58072	0.021124
	B. breve	-2.3092	0.000816

	B. hominis	-1.83007	0.01312
	V. parvula	-1.58527	0.017953
	S. sonnei	-1.48095	0.035288
	*R. mucilaginosa**	1.816241	0.002013
	S. salivarius	2.154098	1.38E-06
	unknown	2.309938	0.001197
	unknown2*	2.415674	0.002465
	S. pneumoniae	2.701254	9.23E-08
	K. michiganensis	2.999949	0.038617
	P. timonensis	3.110778	0.0289
	unknown	3.246996	0.001471
	A. variabilis	3.299921	0.035288
	B. thetaiotaomicron	3.359756	6.06E-05
	*P. distasonis**	3.608224	4.08E-10
	unknown	3.807302	0.015735
	B. animalis	3.86723	0.035288
	*C. aerofaciens**	4.12414	6.12E-13
	C. difficile	4.151631	0.007071
	R. gnavus	4.427971	0.00189
	H. parainfluenzae	4.456899	1.26E-05
	B. faecis	4.471463	0.002036
	P. merdae	5.001877	1.35E-08
	E. hirae	5.321854	3.14E-05
	E. durans	6.285314	4.57E-07
	unknown	7.058885	1.12E-08
	B. catenulatum	7.303239	0.001197
	P. copri	7.491746	7.86E-08
	unknown	7.710544	4.08E-10
	unknown	8.027774	3.77E-09
	A. johnsonii	8.174787	4.08E-10
	B. producta	8.577522	4.15E-09
	I. butyriciproducens	10.53901	2.40E-13
Wilcoxon	unknown1*	-7.92E-05	0.0327
	unknown2*	1.88E-05	0.00592
	*C. aerofaciens**	2.58E-05	0.0323
	*P. distasonis**	5.64E-05	0.0481
	*R. mucilaginosa**	0.0002	0.0468

*Genera identified by ≥2 tests as significant

Unknown1: >99.5% identity to uncultured *Veillonella* spp (clone 59-8-23; accession #AY423748.1; clone EL130, accession #JQ406541.1; clone 59-7-18, accession #AY394004.1; clone 59-7-6, accession #AY423747.1; clone 6BB429, accession #FJ976252.1)
GACGAACGCTGGCGGCGTGCTTAACACATGCAAGTCGAACGGACCGACATGGAAGC
TTGCTTCTATGAAGGTTAGTGGCGAACGGGTGAGTAACGCGTAATCAACCTGCCCAT
CAGAGGGGGACAACAGTTGGAAACGACTGCTAATACCGCATACGATCCAATCTCGG

CATCGGGACTGGATGAAAGGTGGCCTCTACTTGTAAGCTATCGCTGATGGAGGGGA
TTGCGTCTGATTAGCTAGTTGGAGGGGTAACGGCCCACCAAGGCAATGATCAGTAG
CCGGTCTGAGAGGATGAACGGCCACATTGGGACTGAGACACGGCCCAAACTCCTAC
GGGAGGCAGCAGTGGGGAATCTTCCGCAATGGACGAAAGTCTGACGGAGCAACGCC
GCGTGAGTGATGACGGCCTTCGGGTTGTAAAGCTCTGTTAATCGGGACGAATGGCTA
CCATGCGAATAGTTTGGAAGTTTGACGGTACCGGAATAGAAAGCCACGGCTAACTA
CGTGCCAGCAGCCGCGGTAATACGTAGGTGGCAAGCGTTGTCCGGAATTATTGGGC
GTAAAGCGCGCGCAGGCGGATCTGCCAGTCTGTCTTAAAAGTTCGGGGCTCAACCC
CGTGATGGGATGGAAACTACAGATCTAGAGTATCGGAGGGGAAAGTGGAATTCCTA
GTGTAGCGGTGAAATGCGTAGATATTAGGAGGAACACCAGTGGCGAAGGCGACTTT
CTGGACGATCACTGACGCTGAGGCGCGAAAGCCAGGGGAGCGAACGGGATTAGATA
CCCCGGTAGTCCTGGCCGTAAACGATGGGTACTAGGTGTAGGAGGTATCGACCCCTT
CTGTGCCGTAGTTAACGCAGTAAGTACCCCGCCTGGGGAGTACGGCCGCAAGGTTG
AAACTCAAAGGAATTGACGGGGGCCCGCACAAGCGGTGGAGTATGTGGTTTAATTC
GACGCAACGCGAAGAACCTTACCAGGTCTTGACATTGATGGACAGGTCCAGAGATG
GACTCTCTTCTTCGGAAGCCAGAAAACAGGTGGTGCACGGTTGTCGTCAGCTCGTGT
CGTGAGATGTTGGGTTAAGTCCCGCAACGAGCGCAACCCCTATCTTATGTTACCAGC
ACTTCGGGTGGGGACTCATGAGAGACTGCCGCAGACAATGCGGAGGAAGGCGGGG
ATGACGTCAAATCATCATGCCCCTTATGACCTGGGCTACACACGTACTACAATGGAC
GATAACAGAGGGAAGCGAAGCCGCGAGGTGGAGCCAACCCCAGAAACTCGTTCTCA
GTTCGGATTGCAGGCTGCAACTCGCCTGCATGAAGTCGGAATCGCTAGTAATCGCAG
GTCAGCATACTGCGGTGAATACGTTCCCGGGCCTTGTACACACCGCCCGTCACACCA
CGAAAGTCGGAAGTGCCCAAAGCCGGTGGGGTAACCTTCGGGAGCCAGCCGTCTAA
GGTAAAGTCGATGATTGGGGTG

Unknown2: 99.86% identity with *R. mucilaginosa* isolate JCVI-JB-Rm27 (Accession #CP097094.1)
GACGAACGCTGGCGGCGTGCTTAACACATGCAAGTCGAACGATGAAGCCTAGCTTG
CTAGGTGGATTAGTGGCGAACGGGTGAGTAATACGTGAGTAACCTACCTTTAACTCT
GGGATAAGCCTGGGAAACTGGGTCTAATACCGGATACGACCAATCTCCGCATGGGG
TGTTGGTGGAAAGCGTTATGTAGTGGTTATAGATGGGCTCACGGCCTATCAGCTTGT
TGGTGAGGTAACGGCTCACCAAGGCGACGACGGGTAGCCGGCCTGAGAGGGTGACC
GGCCACACTGGGACTGAGACACGGCCCAGACTCCTACGGGAGGCAGCAGTGGGGA
ATATTGCACAATGGGCGCAAGCCTGATGCAGCGACGCCGCGTGAGGGATGACGGCC
TTCGGGTTGTAAACCTCTGTTAGCAGGGAAGAAGAGAAATTGACGGTACCTGCAGA
GAAAGCGCCGGCTAACTACGTGCCAGCAGCCGCGGTAATACGTAGGGCGCGAGCGT
TGTCCGGAATTATTGGGCGTAAAGAGCTTGTAGGCGGTTTGTCGCGTCTGCTGTGAA
AGGCCGGGGCTTAACTCCGTGTATTGCAGTGGGTACGGGCAGACTAGAGTGCAGTA
GGGGAGACTGGAACTCCTGGTGTAGCGGTGGAATGCGCAGATATCAGGAAGAACAC
CGATGGCGAAGGCAGGTCTCTGGGCTGTAACTGACGCTGAGAAGCGAAAGCATGGG
GAGCGAACAGGATTAGATACCCTGGTAGTCCATGCCGTAAACGTTGGGCACTAGGT
GTGGGGGACATTCCACGTTTTCCGCGCCGTAGCTAACGCATTAAGTGCCCCGCCTGG
GGAGTACGGCCGCAAGGCTAAAACTCAAAGAAATTGACGGGGGCCCGCACAAGCG
GCGGAGCATGCGGATTAATTCGATGCAACGCGAAGAACCTTACCAAGGCTTGACAT
ATACTGGACCGCATCAGAGATGGTGTTTCCCTTCGGGGCTGGTATACAGGTGGTGCA
TGGTTGTCGTCAGCTCGTGTCGTGAGATGTTGGGTTAAGTCCCGCAACGAGCGCAAC

CCTCGTTCTATGTTGCCAGCACGTTATGGTGGGGACTCATAGGAGACTGCCGGGGTC
AACTCGGAGGAAGGTGGGGATGACGTCAAATCATCATGCCCCTTATGTCTTGGGCTT
CACGCATGCTACAATGGCCGGTACAGAGGGTTGCGATACTGTGAGGTGGAGCTAAT
CCCTAAAAGCCGGTCTCAGTTCGGATTGGGGTCTGCAACTCGACCCCATGAAGTCGG
AGTCGCTAGTAATCGCAGATCAGCAACGCTGCGGTGAATACGTTCCCGGGCCTTGTA
CACACCGCCCGTCAAGTCACGAAAGTTGGTAACACCCAAAGCCGGTGGCCTAACCT
TTTGGAGGGAGCCGTCTAAGGTGGGATTGGCGATTGGGACT
