# Supplementary material for: The gut microbiome in early life predicts malaria susceptibility
Source: Front Cell Infect Microbiol. 2026 Jun 23;16:1769376. doi: 10.3389/fcimb.2026.1769376 (PMC13337902; doi:10.3389/fcimb.2026.1769376)
Supplement: Supplementary file 3 [file Table3.pdf]

Supplemental methods file 1

Classifier analysis for six week old fecal microbiome

Of the 72 model configurations tested across six taxonomic levels and six algorithms, 50 models (69.4%) successfully converged, while 22 models failed primarily due to insufficient data complexity for certain algorithms. Naive Bayes showed the highest failure rate with all 12 attempts failing, followed by other methods with 2 failures each. The optimal classifier was achieved using k-Nearest Neighbors with feature selection at the Species taxonomic level, demonstrating exceptional performance with 90.0% balanced accuracy, 91.7% overall accuracy, 100% sensitivity, 80% specificity, 87.5% precision, and 93.3% F1 score. This model utilized 144 microbial features selected through Boruta feature selection from the original species-level taxonomic data.

Across all successful models, k-Nearest Neighbors demonstrated superior performance with mean balanced accuracy of 69.3% ± 10.4% (maximum 90.0%) and mean F1 score of 79.0%, followed by SVM (Radial) with 65.6% ± 10.1% balanced accuracy (maximum 80.0%), Random Forest with 59.3% ± 11.9% (maximum 80.0%), SVM (Linear) with 57.0% ± 11.7% (maximum 72.9%), and Logistic Regression with 52.6% ± 8.2% (maximum 71.4%). Feature selection using Boruta significantly improved model performance, with mean balanced accuracy increasing from 58.3% ± 9.8% (maximum 80.0%) without feature selection to 64.4% ± 13.7% (maximum 90.0%) with feature selection. Feature selection proved most beneficial at the Species level, where it enabled the identification of the optimal 144-feature subset that achieved 90% balanced accuracy.

Performance varied across taxonomic levels, with Species level achieving the highest performance (maximum balanced accuracy 90.0% using k-NN with feature selection), followed

by Order and Genus levels (both maximum 80.0%), Phylum level (80.0%), Family level (72.9%), and Class level (72.9%). The confusion matrix analysis revealed that the best model correctly classified 9 out of 12 test samples, with 5 true positives, 4 true negatives, 1 false positive, and 2 false negatives. The overall accuracy was 75% with a Cohen's kappa of 0.50, indicating moderate agreement beyond chance. The 95% confidence interval for accuracy ranged from 43% to 95%, with the accuracy null hypothesis value of 58% and p-value of 0.19. McNemar's test yielded a p-value of 1.00, suggesting no significant difference in marginal frequencies of correct and incorrect classifications.

Classifier analysis for entire infant fecal microbiome data set

The same classifier analysis pipeline was used to determine if a prior malaria infection could be predicted based upon the microbiome. The dataset included 150 samples from 6W, 3M, 6M, and 1Y timepoints from children with known malaria history, classified as either having experienced malaria at some point ("Yes" (susceptible), n=109, 72.7%) or never having had malaria ("Never" (resistant), n=41, 27.3%).

The best performing model achieved a balanced accuracy of 66.6% using Logistic Regression at the Genus taxonomic level without feature selection. This model demonstrated moderate sensitivity (78.6%) and specificity (54.5%), with an overall accuracy of 71.8% and F1-score of 80.0%.

The top-performing models across taxonomic levels showed balanced accuracies ranging from 54.7% to 66.6%. Genus-level Random Forest with feature selection achieved 64.6% balanced accuracy, while Class-level Logistic Regression reached 63.6% balanced accuracy. Species-level analyses using Logistic Regression with feature selection achieved 60.1% balanced

accuracy.

Performance varied significantly across taxonomic levels, with Genus-level classification showing the highest average performance (mean balanced accuracy: 44.4%), followed by Species (43.3%), Class (41.9%), and Order levels (41.7%). Phylum level showed the lowest performance (average 29.9%). Success rates were highest at the Genus, Class, Family, Order, and Species levels (10 successful models each), while Phylum level showed reduced success (7 successful models).

Nineteen microbial features were identified as statistically significant ($p<0.05$) discriminators. The most important features at the Genus level were *Klebsiella* (composite score: 15.09, $p<0.001$), *Rothia* (composite score: 9.39, $p=0.005$), and *Dorea* (composite score: 8.76, $p=0.002$). Additional significant features included *Butyricicoccus* (composite score: 7.06, $p=0.008$) and *Megasphaera* (composite score: 6.79, $p=0.007$).

The best performing model achieved a balanced accuracy of 66.6% using Logistic Regression at the Genus taxonomic level without feature selection during cross-validation. However, critical evaluation of the final test set revealed significant performance limitations. The confusion matrix from the best model's test set performance showed that the model is incapable of prior malaria cases with this dataset. The Kappa score of 0% indicates that the model performs no better than random chance, and the test accuracy of 72% matches exactly the baseline accuracy achieved by always predicting the majority class ("Never"). This could be due to the rapid changes in the gut microbiome during the first year of life.

While cross-validation suggested promising performance (66.6% balanced accuracy, 78.6% sensitivity, 54.5% specificity), the independent test set revealed the model's complete failure to generalize, classifying all samples as resistant. Despite identifying statistically significant

microbial features and achieving seemingly reasonable cross-validation performance, the model failed completely on independent test data.
